# Peppermint extract improves egg production and quality, increases antioxidant capacity, and alters cecal microbiota in late-phase laying hens

**DOI:** 10.3389/fmicb.2023.1252785

**Published:** 2023-09-21

**Authors:** Miaomiao Bai, Hongnan Liu, Yihui Zhang, Shanshan Wang, Yirui Shao, Xia Xiong, Xin Hu, Rongyao Yu, Wei Lan, Yadong Cui, Xiangfeng Kong

**Affiliations:** ^1^Hunan Provincial Key Laboratory of Animal Nutritional Physiology and Metabolic Process; National Engineering Laboratory for Pollution Control and Waste Utilization in Livestock and Poultry Production; Key Laboratory of Agro-ecological Processes in Subtropical Region; Hunan Provincial Engineering Research Center for Healthy Livestock and Poultry Production; Scientific Observing and Experimental Station of Animal Nutrition and Feed Science in South-Central, Ministry of Agriculture, Institute of Subtropical Agriculture, Chinese Academy of Sciences, Changsha, Hunan, China; ^2^College of Biology and Food Engineering, Fuyang Normal University, Fuyang, China

**Keywords:** laying hens, peppermint extract, egg production and quality, antioxidant capacity, cecal microbiota

## Abstract

**Introduction:**

Peppermint contains substantial bioactive ingredients belonging to the phytoestrogens, and its effects on the production of late-laying hens deserve more attention. This study evaluated the effects of dietary peppermint extract (PE) supplementation on egg production and quality, yolk fatty acid composition, antioxidant capacity, and cecal microbiota in late-phase laying hens.

**Method:**

PE powder was identified by UPLC-MS/MS analysis. Two hundred and sixteen laying hens (60 weeks old) were randomly assigned to four treatments, each for 28 days: (i) basal diet (control group, CON); (ii) basal diet + 0.1% PE; (iii) basal diet + 0.2% PE; and (iv) basal diet + 0.4% PE. Egg, serum, and cecal samples were collected for analysis.

**Results:**

Dietary PE supplementation increased the laying rate, serum triglyceride, immunoglobulin G, and total antioxidant capacity, while 0.2 and 0.4% PE supplementation increased eggshell thickness, serum total protein level, and superoxide dismutase activity of laying hens compared with the CON group (*P* < 0.05). PE addition in diets increased the C14:0, C18:3n3, C18:3n6, C23:0, C24:0, and C24:1n9 contents in the yolk. In addition, the egg yolk saturated fatty acid content was higher (*P* < 0.05) in the 0.2 and 0.4% PE groups compared with the CON and 0.1% PE groups. The microbiota analysis revealed that the cecal phylum Proteobacteria was decreased (*P* < 0.05) in the PE-supplemented groups. A total of 0.4% PE supplementation increased the cecal richness of gram-positive bacteria and decreased the richness of gram-negative and potentially pathogenic bacteria compared with the 0.1% PE group (*P* < 0.05). Microbial function prediction analysis showed that the cecal microbiota of the PE group was mainly enriched by fatty acid degradation, fatty acid metabolism, amino sugar metabolism, nucleotide sugar metabolism, and other pathways. Regression analysis suggested that 0.28–0.36% PE supplementation was the optimal level for improving egg production and quality, antioxidant capacity, and yolk fatty acid in late-phase laying hens.

**Discussion:**

Dietary PE supplementation improved egg production and quality (including yolk fatty acid composition) by increasing serum IgG and antioxidant capacity and modulating the intestinal microbiota in late-phase laying hens.

## 1. Introduction

The layer industry contributes to supporting sustainable food sources and is considered an important part of the world's livestock production. The laying hens during the late phase of laying are characterized by declining egg productivity, quality, and immunity, which directly leads to increased mortality and reduced feeding efficiency (van den Brand et al., 2004). In modern layer breeding, the fast reduction in egg production is associated with decreased ovarian function in layer hens after 60 weeks of age, marking the beginning of the late-laying period (Rakonjac et al., 2014; Chang et al., 2017). Since post-production occupies a large part of the entire layer production cycle, improving the performance of laying hens during the late-laying period will have great economic significance.

Metabolic dysfunctions included immunity, oxidation–reduction imbalance, and intestinal microbiota disturbance, resulting in decreased overall health and production efficiency in late-phase laying hens (Rattanawut et al., 2018). As a complex microbial ecosystem, the gut microbiota is inextricably linked with intestinal barrier function, immunity, and metabolic function (Chen et al., 2019). Nutritional intervention can modulate the structure of the intestinal microbiota and then regulate host metabolism and overall health. For example, dietary essential oils could increase feed efficiency and egg quality by regulating microbial composition (Feng et al., 2021; Ding et al., 2022). Moreover, discrepancies between laying hens in peak and late-phase production are mainly associated with lipid metabolism disorders, impairment of antioxidant properties, and energy generation (Wang W. W. et al., 2019). Changes in lipid synthesis and decomposition in laying hens could directly affect egg lipid composition, which in turn reflects human dietary fatty acid composition and body health (Lee et al., 2010). Owing to the restriction on the use of in-feed antibiotics in animal production, finding safe and effective feed additives for antibiotic alternatives is necessary for the laying industry.

Plant extracts, as biologically active compounds, are safe and free of residue and have been widely used as feed additives in poultry production (Adewole et al., 2021). Peppermint (*Mentha piperita L*.), as a traditional medicinal plant, has been widely used in Chinese medicine. Peppermint has the medicinal functions of dispelling wind, cooling, analgesia, and being antibacterial and anti-inflammatory due to its bioactive components (Dorman et al., 2003). Peppermint extract (PE) mainly contains volatile oil, flavonoids, terpenoids, organic acids, and other components (Grigoleit and Grigoleit, 2005). Flavonoids, as the main active ingredient of peppermint leaf, have similar bioactivities with estradiol and are considered important phytoestrogens. In recent years, many studies have confirmed that flavonoids have a remarkable regulatory effect on the gut microbiota composition (Wang et al., 2022; Han et al., 2023). Moreover, flavonoids can promote gut health by reducing pathogens (such as *Proteobacteria, Pseudomonas*, and *Staphylococcus*) and increasing probiotics (such as *Bifidobacteria* and *Lactobacillus*) (Zhang et al., 2019). Peppermint flavone has strong antioxidant, antibacterial, and immune-boosting properties (Kang et al., 2018). Peppermint has recently been considered a feed additive with various application prospects and has been widely studied in poultry production (Abdel-Wareth and Lohakare, 2020). Therefore, we hypothesized that the supplementation of PE in late-phase laying hens' diets would positively affect body metabolism, immunity, and microbial composition, which might subsequently improve egg performance and quality. Thus, the present study aimed to explore the effects of PE on laying performance, egg quality, yolk fatty acid content, and the cecal microbiota of laying hens during the late-laying period.

## 2. Materials and methods

### 2.1. Ethics statement

The animal operating procedures and care standards followed in this study were reviewed and approved by the Animal Protection and Utilization Professional Committee of the Institute of Subtropical Agriculture, Chinese Academy of Science (No. CAS20220120).

### 2.2. Preparation and metabolite analysis of peppermint extract

Dried and cleaned peppermint leaves were obtained from a commercial supply, pulverized, and passed through a 60-mesh sieve. The peppermint powder was mixed with 75% ethanol (V:V = 1:4), soaked, and extracted for 24 h. The mixture was filtered through the gauze under negative pressure. The filtrate was evaporated and concentrated by rotary evaporation at 180 × g and 75°C and freeze-dried at −40°C until completely dehydrated. The obtained PE powder was sealed and stored at 4°C until further use.

The identification and quantification of PE metabolites were performed using an ultra-performance liquid chromatography (UPLC)–tandem mass spectrometry (MS/MS) system. Fifty milligram samples were extracted with 1.2 mL of pre-cooled 70% methanolic aqueous, mixed, and centrifuged at 4°C, 10,000 × g for 10 min. The extract supernatant was filtrated through the 0.22-μm membrane and injected into the UPLC-MS/MS system with the Agilent SB-C18 column (1.8 μm, 2.1 mm × 100 mm, Agilent, CA, United States). Mobile phase A was pure water with 0.1% formic acid, and mobile phase B was acetonitrile with 0.1% formic acid. The gradient program was as follows: 95% A and 5% B at 0 min, then 5% A and 95% B at 0–9 min, and 95% A and 5.0% B at 10–14 min. The flow velocity was 0.35 mL/min, the column oven was 40°C, and the injection volume was 2 μL. The HPLC effluent was connected to an electrospray ionization (ESI)-triple quadrupole-MS system. The identification of metabolites was carried out according to public databases, such as MassBank, METLIN, and MoToDBnd. Metabolite quantification was expressed as a peak area integral based on the multiple reaction monitoring (MRM) analysis.

### 2.3. Bird management and experimental design

Based on a similar body weight, a total of 216 healthy laying hens at 60 weeks old were selected and randomly assigned into four groups. Each group contained nine replicates with six hens per replicate, and two hens were assigned to an individual cage. All hens were adapted for 5 days and fed a basal diet (corn–soybean meal). Four treatment groups included the control group (CON), hens fed the basal diet, and the treatment groups: hens fed the basal diet supplemented with 0.1% PE (LPE group), 0.2% PE (MPE group), or 0.4% PE (HPE group). The feeding trial lasted for 28 days. All hens had free access to water, nipple drinkers, and feed at all times. Temperature, humidity, and lighting were set according to the standardized commercial layer farms. The composition and nutrient levels of the basal diet are presented in Supplementary Table S1.

### 2.4. Sample collection

At the end of the feeding trial, three eggs per replicate (*n* = 27 eggs/group) were collected for egg quality and yolk fatty acid content determination. Nine representative hens per group, close to the average body weight, were selected for sample collection. Blood samples (3 mL) were collected from the wing vein of the chicken and kept in vacuum tubes for 30 min, and then, serum was obtained after centrifugation at 3,000 × g for 10 min at 4°C and stored at −20°C for biochemical indicators and antioxidant index analyses. Chickens were killed by instantaneous cervical dislocation. A total of 20 g cecal contents were collected, immediately frozen in liquid nitrogen, and stored at −80°C for microbiota composition analysis.

### 2.5. Determination of laying performance and egg quality

The feed intake, egg production, and egg weight were recorded daily to calculate the average daily feed intake (ADFI), average egg weight, and feed/egg ratio. Albumen height, yolk color, and Haugh unit were measured using a multi-function egg analyzer (Israel Orka Food Technology Ltd., Ramat Hasharon, Israel). Egg height and width were measured and recorded to calculate the egg shape index (height/width × 100). The thickness of the eggshell was measured using an eggshell thickness gauge (Israel Orka Food Technology Ltd., Ramat Hasharon, Israel). The Egg Force Reader-01 (Israel Orka Food Technology Ltd., Ramat Hasharon, Israel) was used to measure the eggshell strength.

### 2.6. Analysis of serum biochemical parameters

Serum biochemical parameters, including aspartate aminotransferase (AST), alkaline phosphatase (ALP), cholesterol (CHOL), glucose (GLU), high-density lipoprotein-cholesterol (HDL-C), immunoglobulin G (IgG), immunoglobulin M (IgM), low-density lipoprotein-cholesterol (LDL-C), triglyceride (TG), total protein (TP), uric acid (UA), and urea nitrogen (UN) were measured using commercially available kits (Roche, Mannheim, Germany) and the Beckman CX4 automatic biochemical analyzer (cobas c311; Roche Diagnostics GmbH, Mannheim, Germany).

### 2.7. Determination of serum oxidative stress-related indices

The serum total antioxidant capacity (T-AOC), superoxide dismutase (SOD), and glutathione peroxidase (GSH-Px) activities and the level of malondialdehyde (MDA) were assessed by commercially available assay kits (Nanjing Jiancheng Biotechnology Institute, Nanjing, China) using the Microplate Reader Infinite M200 PRO (Tecan, Männedorf, Switzerland) as described previously (Bai et al., 2021).

### 2.8. Determination of fatty acid contents of egg yolk

The fatty acid contents of egg yolks were determined using high-performance liquid chromatography–mass spectrometry (HPLC/MS). In brief, 0.1 g of freeze-dried yolk sample was weighed and homogenized with 800 μL of 50% acetonitrile/water (v/v) for 1 min at room temperature. After centrifuging at 10,000 × g for 15 min at 4°C, 400 μL of supernatant was collected and thoroughly mixed with 200 μL of 3-NPH (200 mM) and 200 μL of EDC (120 mM; containing 6% pyridine and 400 ng/mL of acetic acid D3), respectively. The mixture was homogenized for 1 min, incubated for 1 h at 40°C, and shaken every 5 min. After incubation, the samples were centrifuged at 10,000 × g for 15 min at 4°C, and the supernatants were filtered through a 0.22-μm filter membrane and diluted 10-fold with 50% acetonitrile–water (containing 100 ng/mL of internal standard) for LC-MS/MS (Waters Corp., Milford, MA, USA) analysis. The contents of saturated fatty acid (SFA), monounsaturated fatty acid (MUFA), and polyunsaturated fatty acid (PUFA) were calculated.

### 2.9. Cecal DNA extraction and sequencing

One cecal content sample was selected from each replicate (*n* = 9 per group) for the cecal microbiota study. The total bacterial genomic DNA was extracted from 0.20 g of cecal contents using a commercial DNA Isolation Kit (MO BIO Laboratories, Carlsbad, USA) according to the CTAB/SDA method (Cuiv et al., 2011). 16S rRNA high-throughput sequencing was used to analyze the composition and diversity of the cecal microbiota by the Biomarker Technologies Company (Beijing, China). Polymerase chain reaction (PCR) amplification of the bacterial 16S rRNA gene in the V4 region was amplified using the universal forward primer F (5′-ACTCCTACGGGAGGCAGCA-3′) and the reverse primer R (5′-GGACTACHVGGGTWTCTAAT-3′) (Huang et al., 2020). Obtained sequencing library QC was performed on an Illumina NovaSeq 6000 (Illumina, San Diego, USA) and converted into sequenced reads using base-calling analysis. The unique barcodes were read on Flash software for each sample and merged with acquired splicing sequences as raw tags. All assembled HiSeq sequences were deposited in the NCBI Sequence Read Archive (SRA) under the accession number PRJNA907168.

### 2.10. Sequencing data analysis

The UCHIME (version 4.2) software was used to identify and remove chimeric sequences to obtain effective tags; all high-quality tags (threshold value at 97%) were clustered into operational taxonomic units (OTUs). Based on the UNITE taxonomy database, representative sequences of OTU were annotated and compared with the microbial reference database. The R language tool was used to generate the community structure map at different classification levels. Species abundance tables were generated using the QIIME software. Linear discriminant analysis (LDA) effect size (LEfSe) analysis was performed to reveal the bacterial biomarkers with statistically significant differences among groups. Moreover, PICRUST software was used to predict functional gene composition by comparing species composition information based on 16S sequencing data. The KEGG pathway and function abundances were calculated by OTU abundance according to the KEGG database. The differences in metabolic pathways of functional genes of microbial communities among the four groups were observed by the KEGG metabolic pathway analysis. BugBase was used to predict organism-level microbiome phenotypes (Ward et al., 2017). In addition, Spearman's correlation analysis between cecal microbiota and laying performance, egg quality, serum biochemical and antioxidant indicators, and yolk fatty acid composition was performed in R software (v 3.2.1).

### 2.11. Statistical analysis

All data except microbiota data were analyzed using IBM SPSS 22.0 software (SPSS Inc., USA). Data are presented as means ± SEM. One-way ANOVA and the Tukey test were performed to analyze differences among different treatment groups. The linear and best-fit quadratic models for dietary PE supplementation dose were determined by regressions of estimation curves. Based on 16S rRNA sequencing data, Kruskal–Wallis analysis was used to compare the differences among treatment groups after non-parametric tests. Differences were considered significant if *P* < 0.05, and 0.05 ≤ *P* < 0.10 was considered a trend.

## 3. Results

### 3.1. Principal composition analysis of metabolites in the peppermint extract

The principal compositions of PE metabolites are abundant and complex, including flavonoids, phenolic acids, terpenoids, alkaloids, and tannins. Based on the peak area score, the top 10 substances in each category are listed in Table 1. Flavonoids in PE mainly include chrysosplenetin, 6-C-methylquercetin-3-O-rutinoside, kaempferide, and luteolin-7-O-rutinoside. Phenolic acids in PE include ethyl caffeate, mudanoside A, and 3,4-dimethoxycinnamic acid. Terpenoids in PE contain corosolic acid, jasminoside C, and sugiol. Alkaloids in PE contain yibeinoside A, perlolyrine, and alanine betaine. Lignans and coumarins in PE mainly include 3-hydroxycoumarin, esculetin, and coumarin. Other metabolites also contain tannins (3,3′,4-O-trimethylellagic acid and corilagin), organic acids (2-isopropylmalic acid and L-citramalic acid), lipids (γ-linolenic acid and lysoPC 18:0), amino acids and derivatives [N(6), N(6)-dimethyl-L-lysine, and (3-hydroxypropanoyl)-L-leucine], and nucleotides and derivatives [N-(1-deoxy-1-fructosyl)-leucine and crotonoside].

**Table 1:** Identification of metabolites in peppermint extract.

**Class**	**Compounds**	**Formula**	**Molecular weight (Da)**	**Ionization model**	**Peak area score**
Flavonoids	Chrysosplenetin (5,4′-Dihydroxy-3,6,7,3′-tetramethoxyflavone)	C19H18O8	3.74E+02	[M+H]+	5.51E+07
	6-C-Methylquercetin-3-O-rutinoside	C28H32O16	6.24E+02	[M+H]+	3.62E+07
	Kaempferide (3,5,7-trihydroxy-4′-methoxyflavone)	C16H12O6	3.00E+02	[M-H]-	3.23E+07
	Luteolin-7-O-rutinoside	C27H30O15	5.94E+02	[M+H]+	3.15E+07
	Chrysoeriol; 5,7,4′-trihydroxy-3′-methoxyflavone	C16H12O6	3.00E+02	[M-H]-	3.10E+07
	Gardenin C	C20H20O9	4.04E+02	[M+H]+	3.01E+07
	Diosmetin-7-O-glucuronide	C22H20O12	4.76E+02	[M+H]+	2.87E+07
	Kaempferol-3-O-glucuronide	C21H18O12	4.62E+02	[M-H]-	2.74E+07
	Robinson-7-O-neohesperidin	C28H32O14	5.92E+02	[M+H]+	2.57E+07
	Eupatilin-7-O-glucoside	C24H26O12	5.06E+02	[M+H]+	2.45E+07
Phenolic acids	Ethyl caffeate	C11H12O4	2.08E+02	[M-H]-	5.88E+07
	Mudanoside A	C14H18O9	3.30E+02	[M+H]+	4.34E+07
	3,4-Dimethoxycinnamic acid	C11H12O4	2.08E+02	[M-H]-	4.34E+07
	5′-Glucosyloxyjasmanic acid	C18H28O9	3.88E+02	[M-H]-	2.84E+07
	Glucosyringic acid	C15H20O10	3.60E+02	[M-H]-	2.83E+07
	Methyl coumalate	C7H6O4	1.54E+02	[M-H]-	2.75E+07
	2,5-Dihydroxybenzoic acid; Gentisic Acid	C7H6O4	1.54E+02	[M-H]-	2.59E+07
	2,3-bis[(2e)-3-(3,4-dihydroxyphenyl)prop-2-enoyl]-2,3-dihydroxybutanedioic acid	C22H18O12	4.74E+02	[M-H]-	2.58E+07
	Salvianolic acid A	C26H22O10	4.94E+02	[M-H]-	2.55E+07
	Gallic acid	C7H6O5	1.70E+02	[M-H]-	2.55E+07
Terpenoids	2,3-Dihydroxyurs-12-en-28-oic acid (corosolic acid)	C30H48O4	4.72E+02	[M-H]-	4.37E+07
	Jasminoside C	C16H24O7	3.28E+02	[M+H]+	3.20E+07
	Sugiol	C20H28O2	3.00E+02	[M+H]+	2.60E+07
	3,19-Dihydroxyurs-12-en-28-oic acid (pomolic acid)	C30H48O4	4.72E+02	[M-H]-	2.18E+07
	7-Oxodehydroabietic acid	C20H26O3	3.14E+02	[M+H]+	2.09E+07
	3α,12β,15α,21β,24-pentahydroxyserratane	C30H50O8	5.38E+02	[M+H]+	1.94E+07
	2,3-Dihydroxylup-20(29)-en-28-oic acid (alphitolic acid)	C30H48O4	4.72E+02	[M-H]-	1.90E+07
	2,3-Dihydroxy-12-ursen-28-oic acid	C30H48O4	4.72E+02	[M-H]-	1.83E+07
	5-formyl-2,7-dihydroxy-1-methyl-9,10-dihydrophenanthrene	C16H14O3	2.54E+02	[M+H]+	1.77E+07
	Loliolide	C11H16O3	1.96E+02	[M+H]+	1.67E+07
Alkaloids	Yibeinoside A	C33H53NO7	5.75E+02	[M+H]+	9.68E+07
	Perlolyrine	C16H12N2O2	2.64E+02	[M+H]+	4.35E+07
	Alanine betaine	C5H11NO2	1.17E+02	[M+H]+	2.93E+07
	N-(1-Deoxy-1-fructosyl)aminononanoic acid	C15H29NO7	3.35E+02	[M+H]+	2.89E+07
	(1R,3S)-1-methyl-1,2,3,4-tetrahydro-β-carboline-3-carboxylic acid	C13H14N2O2	2.30E+02	[M+H]+	2.79E+07
	20-Ethyl-4beta-(acetoxymethyl)-7,8-diacetoxy-1alpha,6beta,14alpha,16beta-tetramethoxyaconitane	C31H47NO10	5.93E+02	[M+H]+	2.04E+07
	Stachydrine	C7H13NO2	1.43E+02	[M+H]+	1.86E+07
	Caffeoylcholine	C14H20NO4	2.66E+02	[M]+	1.82E+07
	4-(2-Pyridylazo)-N,N-dimethylaniline	C13H14N4	2.26E+02	[M+H]+	1.59E+07
	2-Amino-5-[(1-carboxy-2-phenylethyl)amino]-5-oxopentanoic acid	C14H18N2O5	2.94E+02	[M+H]+	1.53E+07
Lignans and coumarins	3-Hydroxycoumarin	C9H6O3	1.62E+02	[M-H]-	5.54E+07
	Esculetin (6,7-Dihydroxycoumarin)	C9H6O4	1.78E+02	[M-H]-	5.28E+07
	Coumarin	C9H6O2	1.46E+02	[M+H]+	4.35E+07
	Skimmin (7-hydroxycoumarin-7-O-glucoside)	C15H16O8	3.24E+02	[M+H]+	1.56E+07
	3,4-Dihydro-4-(4′-hydroxyphenyl)-5,7-dihydroxycoumarin	C15H12O5	2.72E+02	[M+H]+	2.09E+07
	Eucommin A	C27H34O12	5.50E+02	[M-H]-	2.08E+07
	Tracheloside	C27H34O12	5.50E+02	[M-H]-	2.01E+07
	Isoscopoletin-β-D-glucoside	C16H18O9	3.54E+02	[M+H]+	1.20E+07
	5′-Methoxymatairesinoside	C27H34O12	5.50E+02	[M-H]-	1.92E+07
	Hydroxymethyl coumarin glucoside	C16H18O8	3.38E+02	[M+H]+	1.50E+07
Others	γ-Linolenic acid	C18H30O2	2.78E+02	[M-H]-	1.32E+08
	N-(1-Deoxy-1-fructosyl)Leucine	C12H23NO7	2.93E+02	[M-H]-	1.15E+08
	LysoPC 18:0	C26H54NO7P	5.23E+02	[M+H]+	3.46E+07
	N(6),N(6)-Dimethyl-L-lysine	C8H18N2O2	1.74E+02	[M+H]+	3.26E+07
	(3-hydroxypropanoyl)-L-leucine	C9H17NO4	2.03E+02	[M-H]-	3.18E+07
	Crotonoside; 2-Hydroxyadenosine	C10H13N5O5	2.83E+02	[M+H]+	2.76E+07
	2-Isopropylmalic Acid	C7H12O5	1.76E+02	[M-H]-	2.70E+07
	L-Citramalic acid	C5H8O5	1.48E+02	[M-H]-	2.34E+07
	3,3′,4-O-Trimethylellagic acid	C17H12O8	3.44E+02	[M+H]+	5.52E+06
	Corilagin	C27H22O18	6.34E+02	[M-H]-	3.07E+06

### 3.2. Peppermint extract increases laying performance in late-phase laying hens

The effects of PE on laying performance in late-phase laying hens are presented in Table 2. Dietary PE supplementation increased the laying rate (*P* < 0.05) compared with the CON group. Moreover, dietary PE supplementation had significant linear and quadratic effects on the laying rate (*P* = 0.004, linear model: Y = 62.621 + 52.67X; *P* = 0.005, quadratic model: Y = 58.67 + 163.32X−265.53X^2^, estimated optimal dose required was 0.31% PE) and feed/egg ratio (*P* = 0.018, linear model: Y = 3.42–2.72X; *P* = 0.024, quadratic model: Y = 3.64–8.43X + 13.90X^2^, estimated optimal dose required was 0.30% PE). There was no difference in ADFI or average egg weight among dietary groups.

**Table 2:** Effects of dietary supplementation with peppermint extract (PE) supplementation on the laying performance of late-laying hens.

**Items**	**Groups** ^**1**^				***P*** **-values**		
	**CON**	**LPE**	**MPE**	**HPE**	**PE**	**Linear**	**Quadratic**
Laying rate, %	61.20 ± 4.78^a^	78.13 ± 3.32^b^	79.63 ± 1.88^b^	76.90 ± 3.74^b^	0.019	0.004	0.014
ADFI, g/d/hen	102.47 ± 1.57	99.14 ± 0.92	99.62 ± 1.01	100.34 ± 1.16	0.217	0.222	0.286
Average egg weight, g	52.13 ± 0.54	52.02 ± 0.46	52.84 ± 0.57	51.15 ± 0.44	0.198	0.398	0.648
Feed/egg ratio	3.35 ± 0.35	2.77 ± 0.14	2.72 ± 0.13	2.67 ± 0.15	0.133	0.018	0.024

1 CON, control group, basal diet; LPE, the control diet + 0.1% PE; MPE, the control diet + 0.2% PE; HPE, the control diet + 0.4% PE. Data are expressed as means ± SEM (*n* = 9). Means within a row with different superscripts are significantly different (*P* < 0.05).

### 3.3. Peppermint extract improves egg quality in late-phase laying hens

The egg quality results of laying hens are listed in Table 3. The supplementation of MPE and HPE increased the shell thickness (*P* < 0.05) compared with the CON and LPE groups. Dietary PE supplementation reduced (*P* < 0.05) the yolk color compared with the CON group. Regression analysis showed that the yolk color intensity decreased quadratically (*P* = 0.046) and significantly fitted a quadratic model: Y = 10.44–13.70X+22.22X^2^, indicating that 0.31% PE resulted in the lowest yolk color. Albumen height, Haugh unit, shell strength, and shape index showed no significant difference among dietary groups.

**Table 3:** Effects of dietary supplementation with peppermint extract (PE) on the egg quality of late-laying hens.

**Items**	**Groups** ^**1**^				***P*** **-values**		
	**CON**	**LPE**	**MPE**	**HPE**	**PE**	**Linear**	**Quadratic**
Egg weight, g	49.76 ± 1.41	52.51 ± 0.59	52.21 ± 1.38	49.95 ± 1.02	0.221	0.944	0.381
Albumen height	5.63 ± 0.30	6.04 ± 0.27	5.33 ± 0.21	5.47 ± 0.04	0.190	0.569	0.657
Yolk color	10.00 ± 0.31^b^	8.86 ± 0.14^a^	8.71 ± 0.18^a^	9.00 ± 0.26^a^	0.002	0.089	0.034
Haugh unit	77.04 ± 1.94	80.69 ± 1.77	76.43 ± 1.04	78.86 ± 1.08	0.211	0.356	0.660
Shell strength, pa	4.14 ± 0.14	4.23 ± 0.24	4.22 ± 0.14	4.03 ± 0.18	0.844	0.713	0.936
Shell thickness, mm	0.37 ± 0.01^a^	0.36 ± 0.01^a^	0.39 ± 0.01^b^	0.39 ± 0.01^b^	0.002	0.057	0.195
Shape index, %	73.98 ± 0.57	75.13 ± 0.01	77.03 ± 1.19	75.81 ± 2.61	0.130	0.894	0.732

1 CON, control group, basal diet; LPE, the control diet + 0.1% PE; MPE, the control diet + 0.2% PE; HPE, the control diet + 0.4% PE. Data are expressed as means ± SEM (*n* = 9). Means within a row with different superscripts are significantly different (*P* < 0.05).

### 3.4. Peppermint extract alters serum biochemical parameters in late-phase laying hens

To evaluate the effects of dietary PE on serum biochemical parameters, blood samples collected on day 29 of the trial were analyzed using an automatic biochemical analyzer (Table 4). Compared with the CON groups, the serum TP level was increased (*P* < 0.05) in the MPE and HPE groups, while TG and IgG levels were increased (*P* < 0.05) in the PE-supplemented groups. In addition, dietary PE-supplemented groups displayed a trend for an increased (*P* = 0.081) serum CHOL level. Regression analysis showed that the serum UA level significantly increased (*P* = 0.034) and fitted a quadratic model: Y = 3.17 + 30.78X−60.75X^2^, indicating that 0.25% PE was the optimal level of supplementation. There were no effects on serum ALP, AST, Glu, HDL-C, IgM, LDL-C, or UN levels among dietary groups.

**Table 4:** Effects of dietary supplementation with peppermint extract (PE) on the serum biochemical indicators of late-laying period hens.

**Items^2^**	**Groups** ^**1**^				***P*** **-values**		
	**CON**	**LPE**	**MPE**	**HPE**	**PE**	**Linear**	**Quadratic**
ALP, U/L	448.00 ± 113.37	351.88 ± 90.94	440.44 ± 74.12	398.00 ± 116.27	0.877	0.685	0.880
AST, U/L	225.29 ± 14.06	248.14 ± 13.27	245.14 ± 12.34	242.88 ± 11.25	0.599	0.684	0.817
CHOL, mmol/L	2.27 ± 0.20	3.69 ± 0.42	4.03 ± 0.75	4.70 ± 0.80	0.081	0.917	0.068
Glu, mmol/L	15.80 ± 0.31	15.29 ± 0.38	14.73 ± 0.34	15.43 ± 0.25	0.180	0.247	0.194
HDL-C, mmol/L	0.91 ± 0.12	0.88 ± 0.12	0.82 ± 0.10	0.75 ± 0.21	0.872	0.555	0.809
IgG, g/L	0.07 ± 0.01^a^	0.09 ± 0.01^b^	0.09 ± 0.01^b^	0.09 ± 0.01^b^	0.002	0.219	0.184
IgM, g/L	0.06 ± 0.01	0.07 ± 0.01	0.06 ± 0.01	0.05 ± 0.01	0.272	0.129	0.250
LDL-C, mmol/L	0.30 ± 0.05	0.40 ± 0.09	0.67 ± 0.25	0.55 ± 0.14	0.371	0.935	0.376
TG, mmol/L	4.01 ± 0.59^a^	7.31 ± 0.64^b^	7.52 ± 1.01^b^	9.33 ± 1.70^b^	0.022	0.323	0.187
TP, g/L	54.33 ± 1.55^a^	58.61 ± 1.92^ab^	61.03 ± 3.01^b^	63.09 ± 1.94^b^	0.050	0.353	0.083
UA, mg/dL	5.23 ± 0.99	5.66 ± 0.71	5.41 ± 0.71	5.39 ± 0.66	0.984	0.325	0.034
UN, mmol/L	1.03 ± 0.16	0.86 ± 0.14	0.83 ± 0.20	0.84 ± 0.16	0.823	0.570	0.758

1 CON, control group, basal diet; LPE, the control diet + 0.1% PE; MPE, the control diet + 0.2% PE; HPE, the control diet + 0.4% PE.

2 ALP, alkaline phosphatase; AST, aspartate aminotransferase; CHOL, cholesterol; Glu, glucose; HDL-C, high-density lipoprotein-cholesterol; IgG, immunoglobulin G; IgM, immunoglobulin M; LDL-C, lipoprotein-cholesterol; TG, triglyceride; TP, total protein; UA, uric acid; UN, urea nitrogen. Data are expressed as means ± SEM (*n* = 9). Means within a row with different superscripts are significantly different (*P* < 0.05).

### 3.5. Peppermint extract improves serum antioxidant indices in late-phase laying hens

The effects of PE supplementation on serum antioxidant indices in late-phase laying hens are presented in Table 5. Dietary PE supplementation increased (*P* < 0.05) the serum T-AOC activity compared with the CON group. Compared with the CON and LPE groups, the serum SOD activity was increased (*P* < 0.05) in the MPE and HPE groups. Moreover, serum SOD activity was higher (*P* < 0.05) in the HPE group compared with the MPE group. Regression analysis showed that dietary PE treatment had significant linear and quadratic effects on the serum activities of T-AOC (*P* = 0.005, linear model: Y = 1.15 + 2.16X; *P* = 0.003, quadratic model: Y = 0.85 + 6.99X−11.25X^2^, estimated optimal dose required was 0.31% PE) and SOD (*P* = 0.001, linear model: Y = −1.44 + 65.38X; *P* = 0.001, quadratic model: Y = 1.84 + 11.95X + 124.45X^2^). There was no difference in the serum GSH-PX activity or MDA level among dietary groups.

**Table 5:** Effects of dietary supplementation with peppermint extract (PE) on the serum antioxidant indices of late-laying period hens.

**Items^2^**	**Groups** ^**1**^				***P*** **-values**		
	**CON**	**LPE**	**MPE**	**HPE**	**PE**	**Linear**	**Quadratic**
GSH-PX, U/mL	350.35 ± 11.63	359.8 ± 11.01	372.98 ± 3.82	364.25 ± 7.95	0.413	0.896	0.167
MDA, nmol/mL	20.75 ± 2.27	17.02 ± 1.89	18.04 ± 1.08	18.31 ± 1.51	0.530	0.968	0.595
SOD, U/mL	2.75 ± 0.31^a^	3.34 ± 0.59^a^	9.91 ± 0.87^b^	26.38 ± 1.70^c^	0.001	0.001	0.001
T-AOC, U/mL	0.72 ± 0.06^a^	1.60 ± 0.14^b^	1.69 ± 0.15^b^	1.86 ± 0.22^b^	0.001	0.005	0.003

1 CON, control group, basal diet; LPE, the control diet + 0.1% PE; MPE, the control diet + 0.2% PE; HPE, the control diet + 0.4% PE.

2 GSH-Px, glutathione peroxidase; MDA, malondialdehyde; T-AOC, total antioxidant capacity; SOD, superoxide dismutase. Data are expressed as means ± SEM (*n* = 9). Means within a row with different superscripts are significantly different (*P* < 0.05).

### 3.6. Peppermint extract increases the fatty acid content of egg yolk in late-phase laying hens

The fatty acid composition results of egg yolk are listed in Table 6. The contents of C14:0, C18:3n3, C18:3n6, C23:0, and C24:0 in the PE-supplemented groups (LPE, MPE, and HPE), C24:1n9 in the MPE group, and C16:1 in the HPE group were increased (*P* < 0.05), whereas the content of C20:3 in the HPE group was decreased (*P* < 0.05) compared with the CON group. Dietary MPE and HPE supplementation increased (*P* < 0.05) the SFA content in the yolk compared with the CON and LPE groups. The C15:0 content in the egg yolk was higher (*P* < 0.05) in the HPE group compared with the CON and LPE groups. Dietary PE supplementation had trends toward increasing the contents of C16:0 (*P* = 0.094), C17:0 (*P* = 0.053), C18:0 (*P* = 0.089), and C20:3n3 (*P* = 0.090) in the egg yolk.

**Table 6:** Effects of dietary supplementation with peppermint extract (PE) on the fatty acid composition of eggs in late-laying period hens.

**Items, ng/mg**	**Groups** ^**1**^				***P*** **-values**		
	**CON**	**LPE**	**MPE**	**HPE**	**PE**	**Linear**	**Quadratic**
C14:0	1.43 ± 0.05^a^	1.63 ± 0.07^b^	1.74 ± 0.08^b^	1.67 ± 0.06^b^	0.012	0.010	0.012
C15:0	0.10 ± 0.02^a^	0.12 ± 0.01^a^	0.17 ± 0.02^ab^	0.20 ± 0.03^b^	0.018	0.001	0.003
C16:0	19.41 ± 0.74	21.74 ± 1.45	23.52 ± 1.73	23.35 ± 1.19	0.094	0.087	0.127
C16:1	3.48 ± 0.20^a^	4.85 ± 0.25^b^	4.28 ± 0.49^ab^	4.80 ± 0.36^b^	0.016	0.236	0.411
C17:0	0.38 ± 0.02	0.46 ± 0.04	0.53 ± 0.05	0.51 ± 0.04	0.053	0.039	0.062
C17:1	0.13 ± 0.03	0.14 ± 0.03	0.13 ± 0.02	0.17 ± 0.04	0.762	0.007	0.026
C18:0	51.30 ± 2.40	42.79 ± 5.36	59.24 ± 7.72	57.04 ± 2.92	0.089	0.147	0.230
C18:1n9c	5.64 ± 0.26	5.88 ± 0.53	6.16 ± 0.69	13.74 ± 7.36	0.409	0.101	0.259
C18:1n9t	6.77 ± 0.28	6.75 ± 0.57	7.21 ± 0.87	7.42 ± 0.50	0.773	0.136	0.337
C18:2n6t	0.16 ± 0.01	0.21 ± 0.02	0.22 ± 0.03	0.22 ± 0.01	0.200	0.182	0.225
C18:2n6c	18.16 ± 1.40	22.41 ± 2.20	20.67 ± 4.79	23.29 ± 1.62	0.547	0.212	0.291
C18:3n3	0.16 ± 0.02^a^	0.29 ± 0.02^b^	0.29 ± 0.04^b^	0.31 ± 0.02^b^	0.002	0.060	0.034
C18:3n6	0.16 ± 0.02^a^	0.31 ± 0.03^b^	0.31 ± 0.05^b^	0.31 ± 0.02^b^	0.002	0.144	0.059
C20:0	13.60 ± 1.03	11.34 ± 1.85	17.30 ± 5.21	21.38 ± 1.27	0.115	0.275	0.262
C20:2	0.38 ± 0.03	0.44 ± 0.08	0.5 ± 0.12	0.64 ± 0.04	0.112	0.020	0.030
C20:3	0.43 ± 0.05^a^	0.64 ± 0.14^ab^	0.61 ± 0.14^ab^	0.93 ± 0.1^b^	0.034	0.012	0.038
C20:3n3	0.61 ± 0.05	0.65 ± 0.14	0.61 ± 0.16	0.99 ± 0.10	0.090	0.025	0.087
C20:4n6	22.45 ± 1.73	24.87 ± 2.77	24.88 ± 4.02	25.15 ± 1.61	0.878	0.417	0.603
C20:5n3	0.11 ± 0.02	0.23 ± 0.04	0.26 ± 0.08	0.24 ± 0.04	0.142	0.087	0.190
C21:0	2.99 ± 0.89	1.95 ± 0.49	1.32 ± 0.16	1.97 ± 0.30	0.199	0.748	0.021
C22:2	0.17 ± 0.02	0.21 ± 0.05	0.23 ± 0.05	0.27 ± 0.06	0.486	0.057	0.162
C22:3	2.05 ± 0.20	2.12 ± 0.36	2.30 ± 0.64	3.37 ± 0.36	0.139	0.057	0.150
C22:4	1.12 ± 0.10	0.98 ± 0.09	1.11 ± 0.15	1.35 ± 0.12	0.177	0.004	0.017
C22:5n-6	0.69 ± 0.12	0.53 ± 0.09	0.91 ± 0.22	0.95 ± 0.12	0.168	0.011	0.039
C22:5n-3	10.82 ± 1.18	9.59 ± 1.47	8.49 ± 2.07	11.44 ± 0.81	0.495	0.325	0.501
C23:0	45.67 ± 3.68^a^	54.89 ± 1.79^b^	55.53 ± 2.02^b^	58.35 ± 2.14^b^	0.007	0.069	0.065
C24:0	13.95 ± 0.7^a^	17.53 ± 0.87^b^	17.31 ± 0.77^b^	17.88 ± 1.23^b^	0.016	0.124	0.161
C24:1n9	9.00 ± 0.29^a^	10.16 ± 0.22^b^	10.49 ± 0.34^b^	10.91 ± 0.39^b^	0.001	0.020	0.016
SFA^2^	156.56 ± 5.01^a^	157.88 ± 8.47^a^	193.26 ± 14.48^b^	192.83 ± 8.12^b^	0.005	0.010	0.021
MUFA^2^	25.02 ± 0.71	27.61 ± 1.34	28.32 ± 1.94	37.04 ± 7.7	0.223	0.077	0.184
PUFA^2^	59.04 ± 4.57	63.44 ± 6.31	66.9 ± 11.64	67.28 ± 4.32	0.796	0.963	0.829
Σn-6	45.47 ± 2.84	48.79 ± 5.35	41.76 ± 3.51	45.77 ± 2.16	0.631	0.826	0.933
Σn-3	12.51 ± 1.03	12.34 ± 1.37	13.39 ± 1.51	12.53 ± 0.85	0.933	0.919	0.921
Σn-6/Σn-3^2^	3.71 ± 0.19	4.06 ± 0.32	4.17 ± 0.17	3.75 ± 0.28	0.516	0.930	0.312

1 CON, control group, basal diet; LPE, the control diet + 0.1% PE; MPE, the control diet + 0.2% PE; HPE, the control diet + 0.4% PE.

2 SFA (saturated fatty acid) = C6:0 + C7:0 + C10:0 + C13:0 + C14:0 + C15:0 + C16:0 + C17:0 + C18:0 + C20:0 + C21:0 + C23:0 + C24:0; MUFA (monounsaturated fatty acid) = C16:1 + C17:1 + C18:1n9c + C18:1n9t + C24:1n9; PUFA (polyunsaturated fatty acid) = C18:2n6t + C18:2n6c + C18:3n3 + C18:3n6 + C20:2 + C20:3 + C20:3n3 + C20:4n6 + C20:5n3 + C22:2 + C22:3 + C22:4 + C22:5n-6 + C22:5n-3; Σn-6/Σn-3 = (C18:2n6t + C18:2n6c + C18:3n6 + C20:4n6 + C22:5n-6)/(C18:3n3 + C20:3n3 + C20:5n3 + C22:5n-3). Data are expressed as means ± SEM (*n* = 9). Means within a row with different superscripts are significantly different (*P* < 0.05).

Regression analysis showed that dietary PE had significant effects on the contents of C14:0 (*P* = 0.010, linear model: Y = 1.44 + 1.02X; *P* = 0.012, quadratic model: Y = 1.33 + 2.79X−4.15X^2^, estimated optimal dose required was 0.34% PE), C15:0 (*P* = 0.001, linear model: Y = 0.08 + 0.44X; *P* = 0.003, quadratic model: Y = 3.09 + 0.3X + 0.33X^2^), C17:1 (*P* = 0.007, linear model: Y = 0.06 + 0.41X; *P* = 0.026, quadratic model: Y = 0.08 + 0.15X + 0.62X^2^), C20:2 (*P* = 0.020, linear model: Y = 0.43 + 0.93X; *P* = 0.030, quadratic model: Y = 0.32 + 2.57X-3.83X^2^, estimated optimal dose required was 0.33% PE), C22:5n-6 (*P* = 0.011, linear model: Y = 0.56 + 1.66X; *P* = 0.039, quadratic model: Y = 0.50 + 2.57X−2.13X^2^, estimated optimal dose required was 0.36% PE), C24:1n9 (*P* = 0.020, linear model: Y = 9.31 + 4.91X; *P* = 0.016, quadratic model: Y = 8.61 + 15.92X−25.75X^2^, estimated optimal dose required was 0.31% PE), and SFA (*P* = 0.010, linear model: Y = 161.10 + 91.31X; *P* = 0.021, quadratic model: Y = 155.41 + 221.83X−314.79X^2^, estimated optimal dose required was 0.35% PE). Moreover, dietary PE supplementation had significant linear effects on the contents of C17:0 (*P* = 0.039, linear model: Y = 0.39 + 0.55X) and C20:3n3 (*P* = 0.025, linear model: Y = 0.62 + 1.39X), while having significant quadratic effects on the contents of C18:3n3 (*P* = 0.034, quadratic model: Y = 0.15 + 1.49X−2.61X^2^, estimated optimal dose required was 0.28% PE) in the egg yolk. Other fatty acid contents of egg yolk showed no significant difference among dietary groups.

### 3.7. Peppermint extract regulates the composition and function prediction of the cecal microbiota

**Figure 1:**
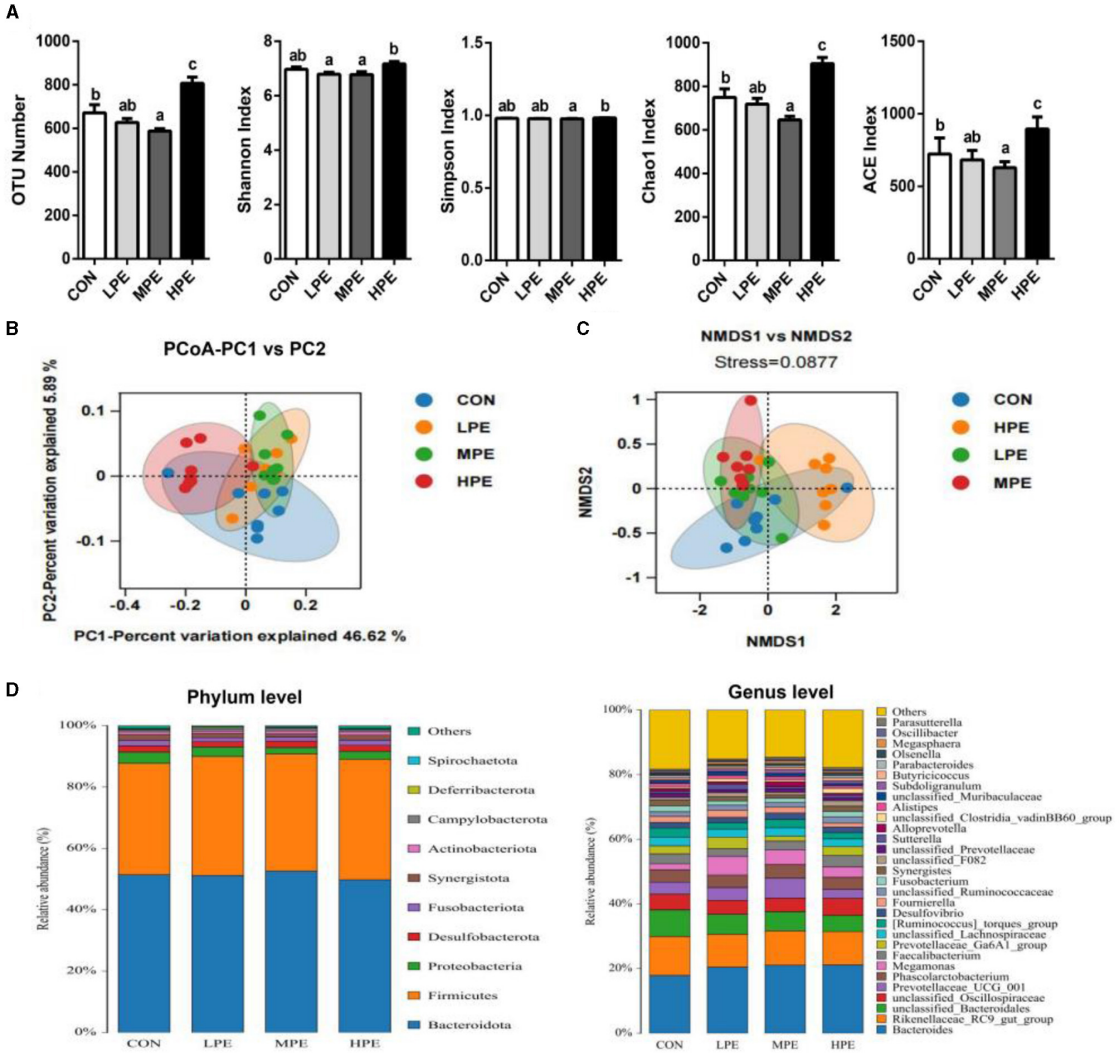
Overall structure and composition of the cecal microbiota after dietary supplementation with peppermint extract (PE). **(A)** The microbial alpha-diversity indices (including OTU number, Shannon, Chao 1, and Simpson indices) of the cecal microbiota. **(B)** Unweighted UniFrac principal coordinate analysis (PCoA) estimates for the cecal microbiota. **(C)** Non-metric multidimensional scaling (NMDS) estimates for cecal microbiota. **(D)** The relative abundance of cecal bacteria at phylum **(left)** and genus **(right)** levels, respectively. CON, control group, basal diet; LPE, the control diet + 0.1% PE; MPE, the control diet + 0.2% PE; HPE, the control diet + 0.4% PE. Data are expressed as means ± SEM (*n* = 8). Means within a row with different superscripts are significantly different (*P* < 0.05).

To assess the cecal microbiota composition in response to PE supplementation, the cecal contents of laying hens were collected for metagenomic sequencing. We evaluated the differences in the alpha-diversity indices among the different treatment groups (Figure 1A). Dietary MPE supplementation decreased (*P* < 0.05) the OTU number, Chao1, and ACE indices, while HPE supplementation increased (*P* < 0.05) the OTU number, Chao1, and ACE indices in the cecal contents of laying hens compared with the CON group. Moreover, the OTU number, Shannon, Simpson, Chao1, and ACE indices were higher (*P* < 0.05) in the HPE group than in the MPE group, while the Shannon index was lower (*P* < 0.05) in the LPE group than in the HPE group.

**Figure 2:**
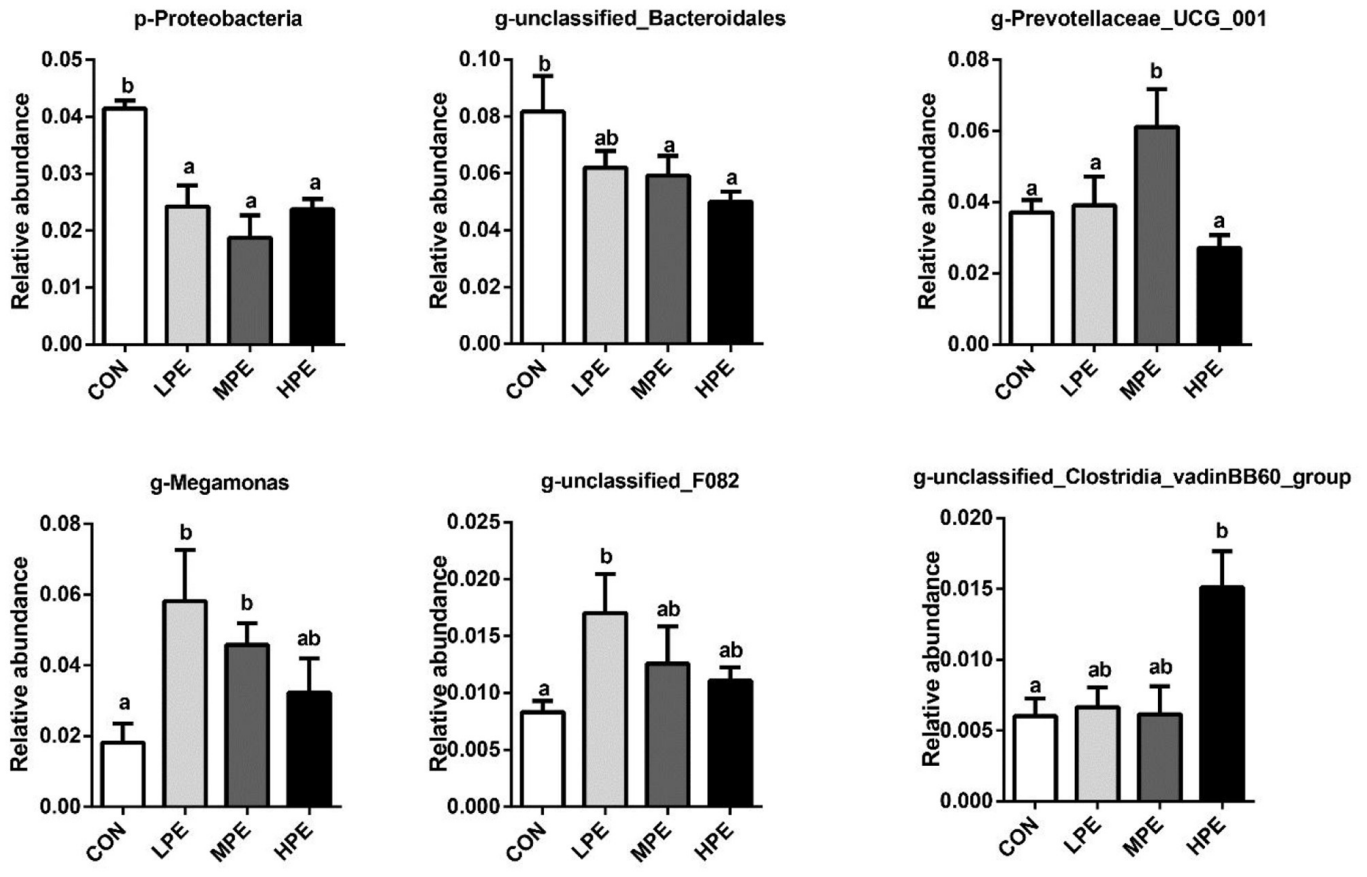
Significantly different cecal microbiota at phylum and genus levels after dietary supplementation with peppermint extract (PE). CON, control group, basal diet; LPE, the control diet + 0.1% PE; MPE, the control diet + 0.2% PE; HPE, the control diet + 0.4% PE. Data are expressed as means ± SEM (*n* = 8). Means within a row with different superscripts are significantly different (*P* < 0.05).

The results of principal coordinate analysis (PCoA, Figure 1B) and non-metric multidimensional scaling (NMDS, Figure 1C) showed that the cecal β-diversity of the CON group intersected and distinguished itself from other PE treatments. The microbiota community composition in cecal contents at the phylum and genus levels is shown in Figure 1D. At the phylum level, the relative abundance of *Bacteroidetes* accounted for at least 50%, followed by *Firmicutes, Proteobacteria*, and *Desulfobacterota*. Taxonomic differences in the microbial community at the phylum and genus levels are shown in Figure 2. Dietary PE supplementation decreased (*P* < 0.05) the relative abundance of *Proteobacteria* at the phylum level. At the genus level, dietary MPE supplementation decreased the relative abundance of *unclassified_Bacteroidales* and increased *Megamonas* compared with the CON group (*P* < 0.05). Dietary LPE supplementation increased the relative abundances of *Megamonas* and *unclassified_F082* (*P* < 0.05), while dietary HPE supplementation decreased *unclassified_Bacteroidales* and increased *unclassified_Clostridia_vadinBB60_group*, when compared with the CON group (*P* < 0.05). Additionally, the relative abundance of *Prevotellaceae_UCG_001* was higher (*P* < 0.05) in the HPE group compared with the other four groups.

**Figure 3:**
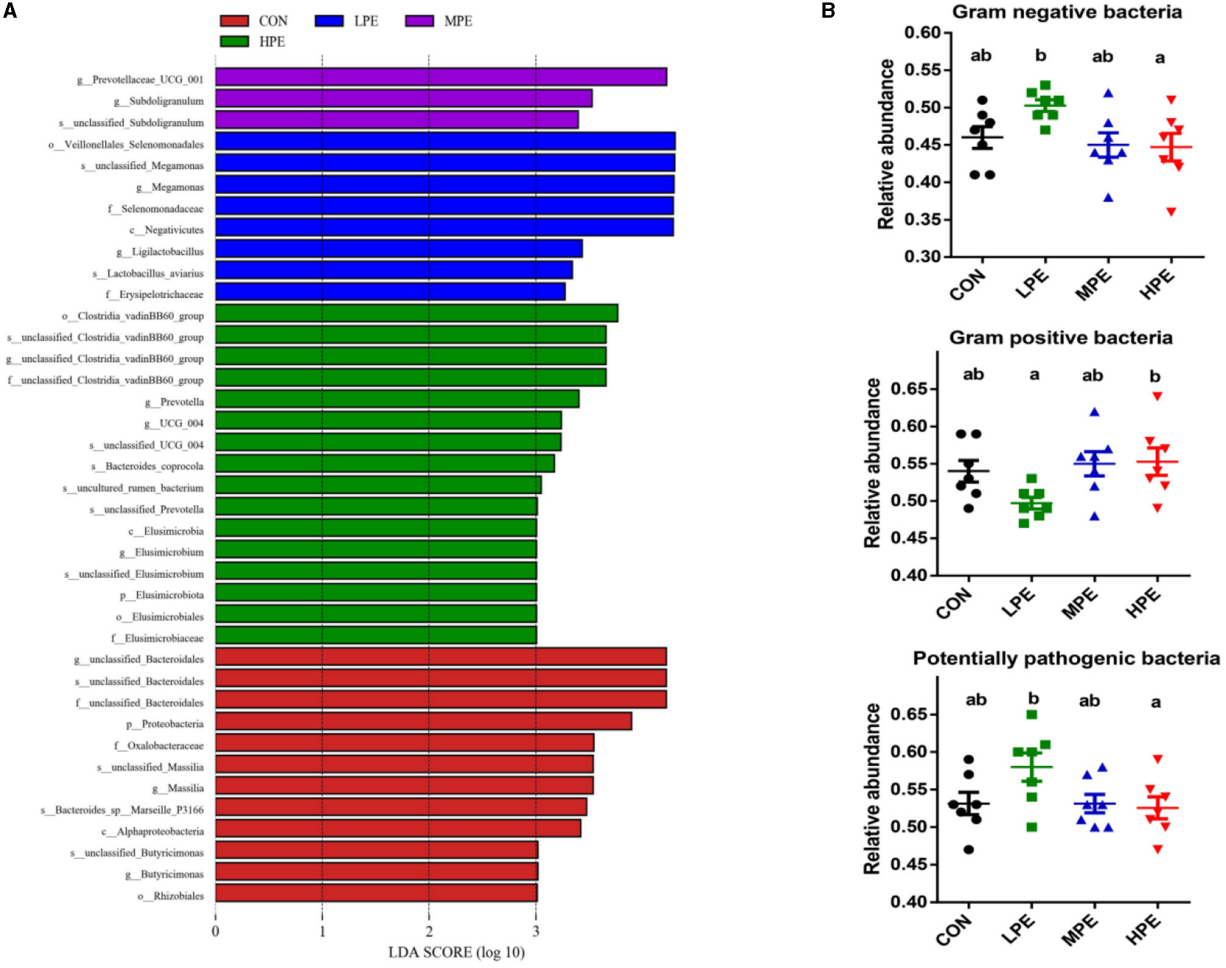
LEfSe analysis and bacterial phenotype prediction results of the cecal microbiota composition after dietary supplementation with peppermint extract (PE). **(A)** Gut microbiota differences based on LDA score (log > 3); **(B)** The metabolic phenotype prediction (gram-negative, gram-positive, and potential pathogenic bacteria) were compared using BugBase analysis. CON, control group, basal diet; LPE, the control diet + 0.1% PE; MPE, the control diet + 0.2% PE; HPE, the control diet + 0.4% PE. Data are expressed as means ± SEM (*n* = 8). Means within a row with different superscripts are significantly different (*P* < 0.05).

As shown in Figure 3A, LEfSe analysis showed that 39 biomarkers were significantly different among the four treatment groups, including 12, 8, 3, and 16 biomarkers in the CON, LPE, MPE, and HPE groups, respectively. BugBase analysis was used to estimate the metabolic phenotype prediction (Figure 3B). The relative abundances of gram-negative and potentially pathogenic bacteria were downregulated, while gram-positive bacteria were upregulated in the HPE group compared with the LPE group (*P* < 0.05).

**Figure 4:**
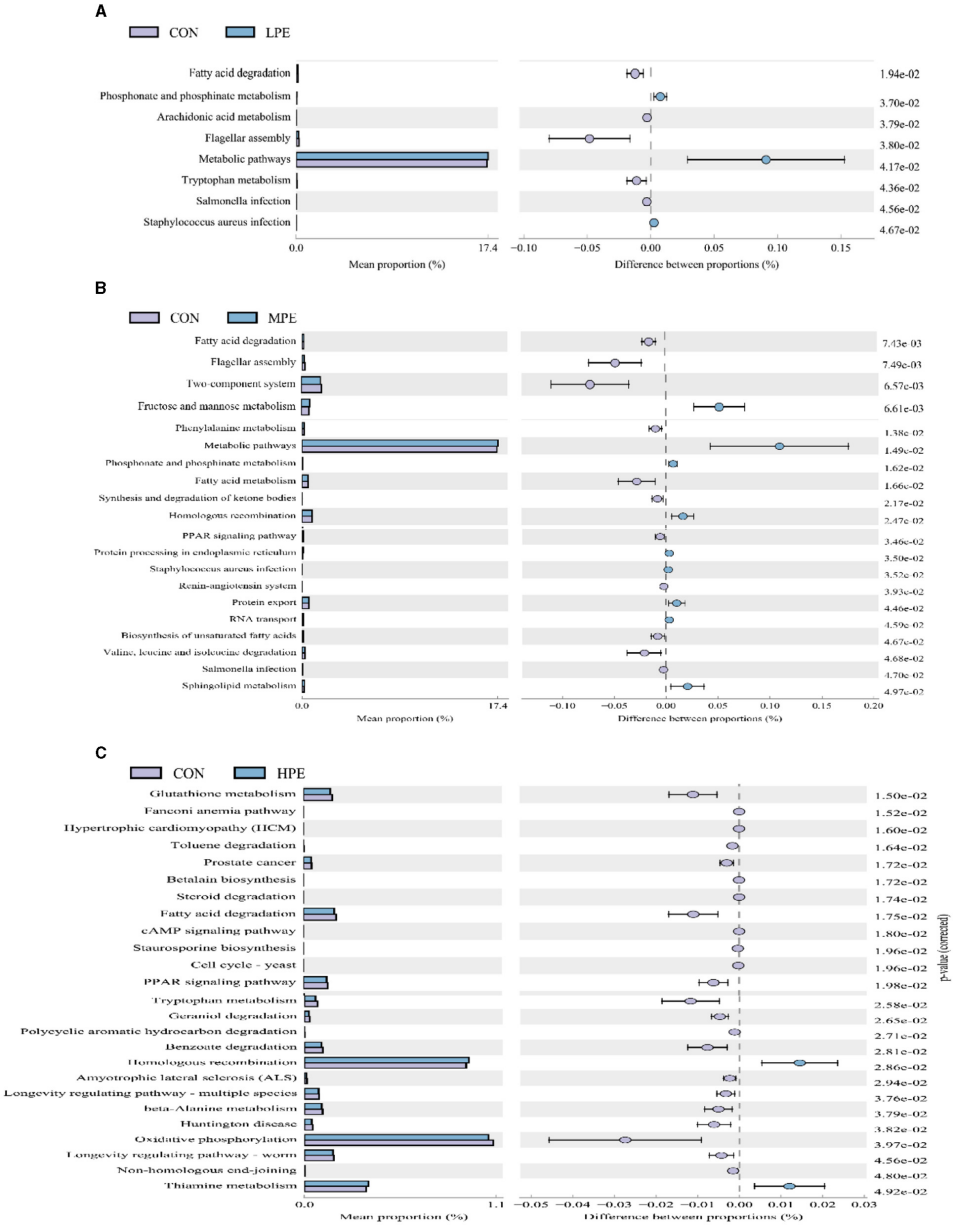
Differences in the metabolic functions of the cecal microbiota after dietary supplementation with peppermint extract (PE). **(A)** *T-*test bar plot of significantly different metabolic pathways between CON and LPE groups; **(B)** *T-*test bar plot of significantly different metabolic pathways between CON and MPE groups; **(C)** *T*-test bar plot of significantly different metabolic pathways between CON and HPE groups. CON, control group, basal diet; LPE, the control diet + 0.1% PE; MPE, the control diet + 0.2% PE; HPE, the control diet + 0.4% PE. Data are expressed as means ± SEM (*n* = 8).

Significant differences in the KEGG pathways at level 3 based on PICRUSt gene prediction information between the two groups are shown in Figure 4. Microbial gene functions related to metabolic pathways, including phosphonate and phosphinate metabolism and metabolic pathways, were upregulated, while fatty acid degradation and flagellar assembly were downregulated in the LPE group (*P* < 0.05; Figure 4A) compared with the CON group. Metabolic pathways, fructose and mannose metabolism, amino sugar and nucleotide sugar metabolism, and sphingolipid metabolism were upregulated (*P* < 0.05), while fatty acid degradation and fatty acid metabolism were downregulating (*P* < 0.05) in the MPE group compared with the CON group (Figure 4B). Dietary HPE supplementation upregulated (*P* < 0.05) several metabolic pathways, including homologous recombination and thiamine metabolism, while downregulated (*P* < 0.05) glutathione metabolism, fatty acid degradation, the PPAR signaling pathway, and benzoate degradation compared to the CON group (Figure 4C).

**Figure 5:**
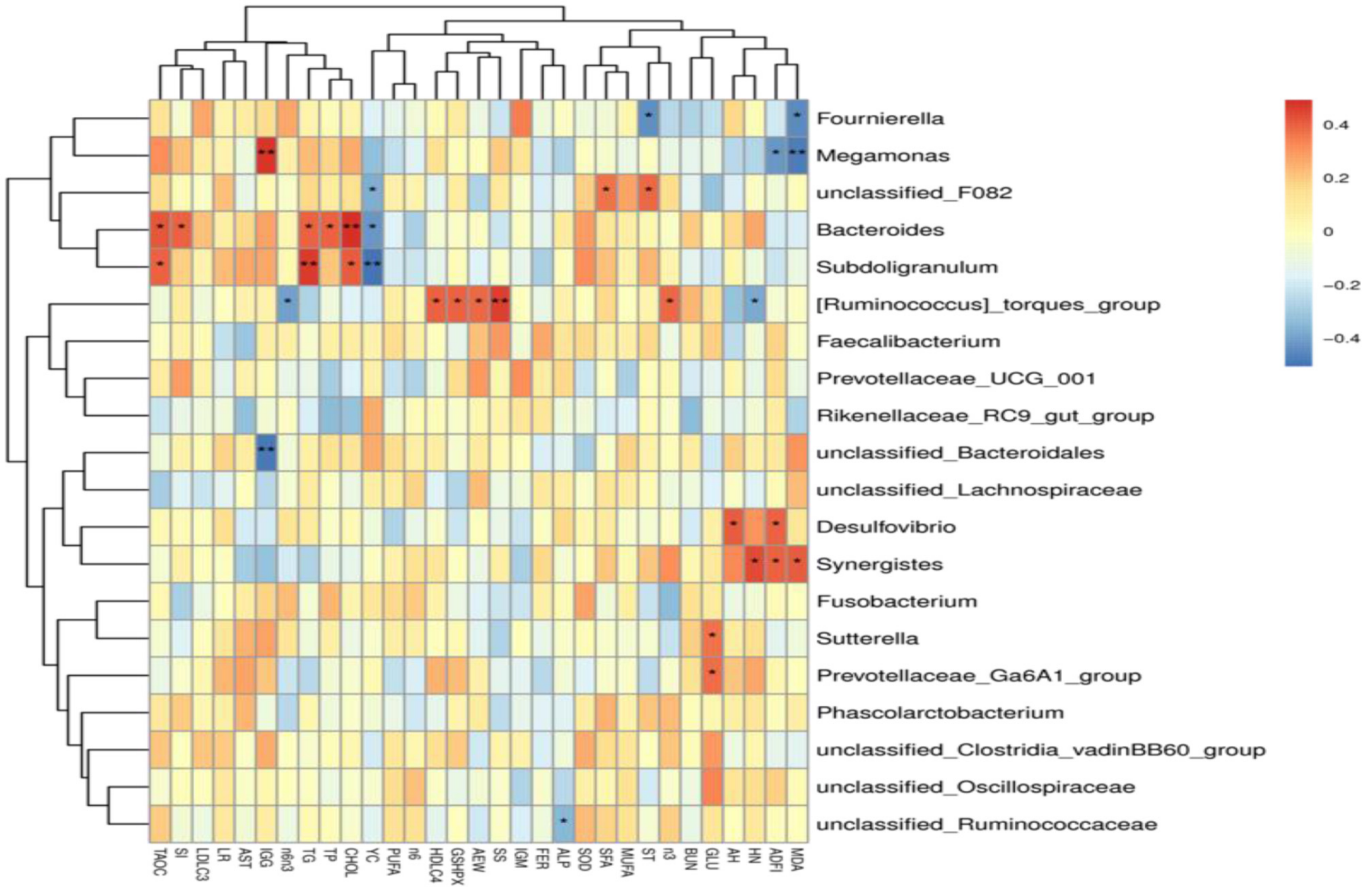
Heatmap of Spearman's correlations analysis. Spearman's correlation coefficients between the major genera and laying performance, egg quality, serum biochemical and antioxidant indicators, and yolk fatty acid composition. ^*^P < 0.05, ^**^P < 0.01.

The results of Spearman's correlation analysis between the major genera and laying performance, egg quality, serum biochemical and antioxidant indicators, and yolk fatty acid composition were presented with a heatmap (Figure 5). *Megamonas* had significant positive relations with serum IgG level (*P* < 0.05) and was negatively correlated with ADFI, Haugh unit, and serum MDA level (*P* < 0.05). *Fournierella* showed significant negative correlations with serum MDA levels and shell thickness (*P* < 0.05). *Bacteroides* had significant positive correlations with serum T-AOC activity, shape index, serum TP, TG, and CHOL levels (*P* < 0.05) and were negatively correlated with yolk color (*P* < 0.05). *[Ruminococcus]_torques_group* had significant positive relations with average egg weight, serum HDL-C level, GSH-Px activity, and shell strength (*P* < 0.05), and was negatively correlated with Haugh unit and a ratio of Σn-6/Σn-3 (*P* < 0.05). *Desulfovibrio* showed significant negative correlations with ADFI and albumen height (*P* < 0.05), and *Synergistes* had significant positive relations with ADFI, Haugh unit, and serum MDA level (*P* < 0.05). *Sutterella* and *Prevotellaceae_Ga6A1_group* were positively correlated with serum GLU levels (*P* < 0.05). *unclassified_Ruminococcaceae* was negatively correlated with serum ALP activity (*P* < 0.05).

## 4. Discussion

In recent years, poultry producers have sought solutions to enhance gut health, improve egg production performance, and extend the laying period of late-phase laying hens. The beneficial effects of medicinal plants as feed additives during the late-phase laying period of hens have been reported earlier (An et al., 2019; Peng et al., 2022). The addition of plant extracts, as feed additives in poultry diets, effectively improves poultry health and performance during the late-laying period. Therefore, the present study was conducted to evaluate the beneficial effects of PE supplementation on late-phase laying hens. Our findings indicated that dietary PE supplementation increased the laying performance and egg quality of late-phase laying hens by improving the antioxidant capacity and altering the cecal microflora composition.

Peppermint, as a traditional herb, has been found to have beneficial effects on laying performance. Abdel-Wareth and Lohakare (2014) reported that diets supplemented with peppermint leaves could improve the feed conversion ratio and egg production of laying hens during the late-laying period. It has also been found that 296 mg/kg of peppermint oil positively affected feed intake, egg production, and the conversion ratio of laying hens (Abdel-Wareth and Lohakare, 2020), whereas, in the present study, we found that dietary PE supplementation could improve egg production but did not affect the feed efficiency of laying hens during the late-phase laying period. Rahman et al. (2021) also revealed that an ethanolic extract of peppermint had no detectable differences in the feed conversion ratio of laying hens. Our findings indicated that dietary PE supplementation of 0.30 and 0.37% was required for optimal laying performance in late-phase laying hens. The active components of peppermint, such as menthol, menthone, and linalool, are considered the key components for exerting beneficial effects, including enhancing nutrient utilization, antioxidant capacity, and intestinal health. However, these inconsistencies in the results might be correlated with the feeding cycle, active ingredients, and supplementing dose.

Egg quality, as an important evaluation indicator for both producers and consumers, is closely associated with economic profitability in the egg-food industry (Lordelo et al., 2017). Eggshell thickness is an important indicator of egg quality, and high eggshell thickness contributes to protecting the contents of the egg from mechanical impact and microbacterial invasion (Nys et al., 2004). In the present study, dietary supplementation of 0.2 and 0.4% PE in late-phase laying hens increased eggshell thickness, indicating that dietary PE can enhance the stability and safety of eggs. In accordance with our results, dried peppermint leaves or peppermint oil treatment could significantly increase the eggshell thickness of laying hens (Akbari et al., 2016; Ding et al., 2017). Herbal essential oils have been found to improve eggshell quality by enhancing uterine health and promoting calcium deposition in laying hens (Nadia et al., 2008; Wang H. et al., 2019). Moreover, our findings also showed that dietary PE supplementation decreased egg yolk color. Yolk color is one of the important indices for evaluating egg quality because it determines carotenoid deposition in the egg yolk. Once dietary calcium or vitamin A levels are too high, egg yolk pigmentation will be severely limited (Chen and Bailey, 1988). However, Cetingul et al. (2012) reported that dietary peppermint supplementation had positive impacts on yolk color in laying quails. Therefore, the double-edged effects of PE on egg yolk color pigmentation need further investigation.

Serum biochemical parameters reflect the health and metabolic status of birds. Several studies demonstrated that peppermint oil supplementation could linearly increase the TP level and decrease the CHOL level in the serum of laying hens (Bozkurt et al., 2012; Abdel-Wareth and Lohakare, 2020). In addition, Abdel-Wareth and Lohakare (2014) also demonstrated that the serum TP level increased significantly and the CHOL level decreased in laying hens fed a diet containing 2% peppermint leaf powder. Consistent with our findings, dietary PE supplementation resulted in an increase in the serum TP level in late-phase laying hens. High levels of serum TP reflect that the deposition of body protein is at a high level, whereas, in the present study, the serum TG and CHOL levels increased with the PE treatment. We hypothesize that the effect of dietary PE on the regulation of lipid metabolism is a complex network process due to its various active components. IgG, one of the main types of immunoglobulins, plays a vital role in immune regulation in poultry (Kim and Lillehoj, 2019). Dietary PE supplementation effectively increased the serum IgG level, indicating that dietary PE could enhance the immunity of hens during the late-phase laying period. In contrast, Rahman et al. (2021) reported that 200 mg/kg PE supplementation caused a decrease in serum IgG levels in laying hens. The conflicting effects on serum biochemical markers of peppermint supplementation among several studies were possibly due to the chemical composition and concentration of active ingredients; however, further in-depth studies are necessary to reveal the exact mechanism.

The decline in laying performance and egg quality during the late-phase laying period is mainly associated with the accumulation of oxidative stress in long-term laying production (Liu et al., 2018). Numerous studies have demonstrated that high levels of reactive oxygen species accumulated in the ovaries of aged chickens resulted in oxidative stress, sinus follicular atresia, and granulosa cell apoptosis, which in turn, decreased reproductive capacity (Agarwal et al., 2012; Rizzo et al., 2012). Therefore, enhancing the antioxidant capacity of hens during the late-phase laying period is an important measure to improve the laying performance of laying hens. In recent years, various natural herb extracts such as tea polyphenols (Zhou et al., 2021), *Scutellaria* extracts (Varmuzova et al., 2015), and hesperidin (Khedr, 2015) have been applied to reduce ovarian oxidative stress and improve reproductive performance in laying hens. Dorman et al. (2009) found that phenols and flavonoids, as the main active components of PE, could effectively scavenge free radicals and protect tissues from oxidative stress damage. In the present study, the serum activities of T-AOC and SOD were increased in late-phase laying hens supplemented with PE. T-AOC is the total antioxidant level of various antioxidant substances and antioxidant enzymes. SOD is one of the most representative enzymes in the antioxidant system, which can eliminate superoxide anion free radicals (O^−2^) to protect cells from damage. Collectively, dietary PE can improve the serum activity of antioxidant enzymes to effectively ameliorate oxidative stress. The amelioration of oxidative stress by PE may contribute to improving ovarian function and increasing the laying performance and egg quality of hens during the late-phase laying period.

The fatty acid composition of egg yolks is also an important part of assessing egg quality and nutritional value. The changes in lipid metabolism in laying hens can directly affect yolk lipid composition, and nutritional regulation is one of the main pathways to changing the fatty acid contents. Previous studies have confirmed that the dietary addition of plant extract could change fatty acid composition in laying hens (Kaya et al., 2013; Duan et al., 2022). In the present study, dietary PE supplementation increased SFA and PUFA contents in the egg yolk, suggesting that PE could regulate egg fat deposition. As a complete food source for human nutrition, the fatty acid composition of eggs is closely related to human health (Miranda et al., 2015). Studies have found that dietary SFA may increase the plasma LDL-C level and contribute to cardiovascular disease development (Griel and Kris-Etherton, 2006). Intake of dietary PUFA could decrease blood CHOL and LDL-C levels and thus reduce the incidence of cardiovascular disease (Masquio et al., 2015). However, some SFAs have potential physiological effects and are indispensable for maintaining normal physiological functions in the body. Especially, C18:0 can reduce the absorption of intestinal cholesterol and reduce the occurrence of cardiovascular diseases, which is similar to n-3 PUFA (Moyano et al., 2013). Our findings indicated that PE supplementation increased the contents of C18:0, C18:3n3, and C18:3n6 in the egg yolk. C18:3n3, as an important essential fatty acid and a precursor to the synthesis of n-3 PUFA, can promote brain and retina development (Calder, 2015). C18:3n6 can be converted to C20:4n6 by metabolic pathways and contributes to improving vision and memory, lowering plasma cholesterol, and regulating nerves (Masquio et al., 2015). Moreover, we also found that dietary PE supplementation had no impact on the n-6/n-3 ratio in the egg. In agreement with our findings, Karadaolu et al. (2018) also reported that n-6/n-3 ratios were not affected in laying hens when supplemented with peppermint oil in the drinking water. Taken together, dietary PE supplementation in late-phase laying hens' diets could improve the fatty acid composition of eggs, which was consistent with the increased serum TG and CHOL levels. Therefore, our findings suggest that the fatty acid content in egg yolk was optimized at 0.21 to 0.36% of PE. To our knowledge, the research on the effects of PE on the fatty acid composition of egg yolk is limited, and further studies are needed to support more detailed information on this topic.

The gut microbiota, a complex microbial ecosystem, is closely linked to immunity and nutrient absorption (Zhao et al., 2019). Structural changes in the intestinal microbiota have been described as important biomarkers to assess the influence of a specific dietary component (Hughes et al., 2019). In the present study, dietary PE supplementation altered the intestinal microbiota in laying hens during the late-phase laying period, including a decrease in the abundance of *Proteobacteria*. *Proteobacteria* are characterized by adherent and invasive properties, leading to pro-inflammatory responses and even the development of inflammatory bowel diseases (Mukhopadhya et al., 2012). An increased abundance of *Proteobacteria* has been used as a potential diagnostic marker to assess the occurrence of prevalence (Shin et al., 2015). Thus, dietary PE may reduce the occurrence of diseases in laying hens during the late-phase period by inhibiting intestinal pathogens. In addition, dietary 0.2% PE supplementation decreased the cecal microbial richness (Chao1 and ACE indices) and the relative abundance of *Unclassified_Bacteroidales*, whereas it increased the relative abundance of *Megamonas*. The 0.4% PE diet increased the cecal microbial richness and the relative abundances of *Unclassi-fied_Clostridia_vadinBB60_group* and *Prevotellaceae_UCG_001*, whereas it decreased the relative abundance of *Unclassified_Bacteroidales*. A recent study reported a significant positive correlation between *Unclassified_Bacteroidales* abundance and pro-inflammatory cytokines levels (Sun et al., 2022). *Megamonas*, belonging to the phylum Firmicutes, could ferment various carbohydrates to produce acetic, propionic, and lactic acids, which were negatively associated with the incidence of enteritis (Yachida et al., 2019). *Prevotellace-ae_UCG_001* is often considered a bacterium associated with a healthy plant-based diet, acting as a probiotic in the human body (Ley, 2016). Changes in the abundance of immune-associated flora (*Proteobacteria* and *Unclassified_Bacteroidales*) in the cecum may be the key to improving serum IgG levels. Collectively, the intervention of dietary PE might play a critical role in improving intestinal health by altering the cecal microbiota of laying hens during the late-phase laying period.

Based on the prediction of microbial functions, the present study first revealed the microbial phenotypes in late-phase laying hens treated with different PE levels. The 0.4% PE diet reduces the proliferation of gram-negative and potentially pathogenic bacteria but improves the proliferation of gram-positive bacteria. These findings are consistent with changes in the structure of the cecal microflora caused by dietary PE supplementation in the present study. In addition, the analysis of metabolic function showed that dietary PE supplementation decreased fatty acid metabolism, fatty acid degradation, and PPAR signaling pathways, which is partly explained by the fact that dietary PE can promote fatty acid deposition in the egg yolk of laying hens during the late-phase laying period. However, the specific pathway regulation by PE diets of fatty acid metabolism in late-phase laying hens needs further verification.

## 5. Conclusion

In summary, this study provides compelling evidence that PE is effective in improving egg production and quality in late-phase laying hens through increasing antioxidant capacity, regulating metabolism level, and altering cecal microbial communities, which are associated with the regulation of laying rate, eggshell thickness, serum IgG, antioxidant enzyme activities, the contents of C18:3n3 and C18:3n6 in the egg yolk, and microbial richness, metabolic phenotypes, and functions. These findings indicate that PE has the potential to be an egg production promoter for laying hens during the late-phase laying period. Based on the regression analysis, the optimal dosage of peppermint extract as a feed additive in late-phase laying hens was 0.28 to 0.36%. This study highlights the importance of PE supplementation during the late-phase laying period.

## Data availability statement

The datasets presented in this study can be found in online repositories. The names of the repository/repositories and accession number(s) can be found in the article/Supplementary material.

## Ethics statement

The animal operating procedures and care standards followed in this study were reviewed and approved by the Animal Protection and Utilization Professional Committee of the Institute of Subtropical Agriculture, Chinese Academy of Science (No. CAS20220120). The study was conducted in accordance with the local legislation and institutional requirements.

## Author contributions

HL and XK: conceptualization and resources. WL and HL: methodology. MB, YZ, and SW: formal analysis. MB and XH: investigation. YS and RY: data curation. MB: writing—original draft preparation. HL, XX, and YC: writing—reviewing and editing. HL, YC, and XK: funding acquisition. All authors have read and agreed to the published version of the manuscript.

## References

[B1] Abdel-Wareth A. A. A. Lohakare J. D. (2014). Effect of dietary supplementation of peppermint on performance, egg quality, and serum metabolic profile of Hy-Line Brown hens during the late laying period. Anim. Feed Sci. Tech. 197, 114–120. 10.1016/j.anifeedsci.2014.07.007

[B2] Abdel-Wareth A. A. A. Lohakare J. D. (2020). Productive performance, egg quality, nutrients digestibility, and physiological response of bovans brown hens fed various dietary inclusion levels of peppermint oil. Anim. Feed Sci. Tech. 267, 114554. 10.1016/j.anifeedsci.2020.114554

[B3] Adewole D. I. Oladokun S. Santin E. (2021). Effect of organic acids-essential oils blend and oat fiber combination on broiler chicken growth performance, blood parameters, and intestinal health. Anim. Nutr. 7, 1039–1051. 10.1016/j.aninu.2021.02.001 34738034 PMC8546314

[B4] Agarwal A. Aponte-Mellado A. Premkumar B. J. Shaman A. Gupta S. (2012). The effects of oxidative stress on female reproduction: a review. Reprod. Biol. Endocrinol. 10, 49. 10.1186/1477-7827-10-49 22748101 PMC3527168

[B5] Akbari M. Torki M. Kaviani K. (2016). Single and combined effects of peppermint and thyme essential oils on productive performance, egg quality traits, and blood parameters of laying hens reared under cold stress condition (6.8 +/– 3 A degrees C). Int. J. Biometeorol. 60, 447–454. 10.1007/s00484-015-1042-6 26238513

[B6] An S. Y. Liu G. Z. Guo X.;, An, Y. H. Wang R. Y. (2019). Ginger extract enhances antioxidant ability and immunity of layers. Anim. Nutr. 5, 407–409. 10.1016/j.aninu.2019.05.003 31890918 PMC6920395

[B7] Bai M. M. Liu H. N. Wang S. S. Shu Q. Y. Xu K. Zhou J.. (2021). Dietary moutan cortex radicis improves serum antioxidant capacity and intestinal immunity and alters colonic microbiota in weaned piglets. Front. Nutr. 8, 679129. 10.3389/fnut.2021.679129 34222303 PMC8247480

[B8] Bozkurt M. Tokusoglu O. Kucukyilmaz K. Aksit H. Cabuk M. Catli A. U.. (2012). Effects of dietary mannan oligosaccharide and herbal essential oil blend supplementation on performance and oxidative stability of eggs and liver in laying hens. Italy J. Anim. Sci. 11, 1056–1060. 10.4081/ijas.2012.e41

[B9] Calder P. C. (2015). Very long chain omega-3 (n-3) fatty acids and human health. Eur. J. Lipid. Sci. Technol. 116, 1280–1300. 10.1002/ejlt.201400025

[B10] Cetingul I. S. Ismail B. Akkaya A. B. Cangir U. Mehmet Y. (2012). Effect of peppermint (mentha piperita) on performance, hatchability and egg quality parameters of laying quails (coturnix coturnix japonica). J. Anim. Vet. Adv. 7, 1489–1494. 10.2460/javma.233.9.1453 18980501

[B11] Chang Y. Jinghai F. J. H. Zhang M. H. Jiang L. W. Zhai L. L. (2017). Effects of massive ovulation on oxidation state and function of the ovaries in laying hens. Turk. J. Vet. Anim. Sci. 41,161–166. 10.3906/vet-1603-57

[B12] Chen B. H. Bailey C. A. (1988). Effect of turf bermudagrass meal on egg production, feed utilization, yolk color, and egg weight. Poult. Sci. 67, 1154–1156. 10.3382/ps.0671154

[B13] Chen M. X. Wang S. Y. Kuo C. H. Tsai I. L. (2019). Metabolome analysis for investigating host-gut microbiota interactions. J. Formos. Med. Assoc. 118, S10–S22. 10.1016/j.jfma.2018.09.007 30269936

[B14] Cuiv P. O. de Carcer D. A. Jones M. Klaassens E. S. Worthley D. L. Whitehall V. L. J.. (2011). The effects from DNA extraction methods on the evaluation of microbial diversity associated with human colonic tissue. Microbial. Ecol. 61, 353–362. 10.1007/s00248-010-9771-x 21153634

[B15] Ding X. M. Yang Y. Su Z. W. Zhang K. Y. (2017). Effects of essential oils on performance, egg quality, nutrient digestibility and yolk fatty acid profile in laying hens. Anim. Nutr. 3, 127–131. 10.1016/j.aninu.2017.03.005 29767138 PMC5941116

[B16] Ding Y. A. Hu Y. Yao X. F. He Y. C. Chen J. L. Wu J.. (2022). Dietary essential oils improves the growth performance, antioxidant properties and intestinal permeability by inhibiting bacterial proliferation, and altering the gut microbiota of yellow-feather broilers. Poult. Sci. 101, 102087. 10.1016/j.psj.2022.102087 36095866 PMC9472070

[B17] Dorman H. J. D. Koşar M. Başer K. Hiltunen R. (2009). Phenolic profile and antioxidant evaluation of Mentha x piperita L. (peppermint) extracts. Nat. Prod. Commun. 4, 535–542. 10.1177/1934578X0900400419 19476001

[B18] Dorman H. J. D. Kosar M. Kahlos K. Holm Y. Hiltunen R. (2003). Antioxidant properties and composition of aqueous extracts from Mentha species, hybrids, varieties, and cultivars. J. Agri. Food. Chem. 51, 4563–4569. 10.1021/jf034108k 14705878

[B19] Duan S. H. Li Z. Q. Fan Z. Z. Qin M. R. Yu X. X. Li L. A. (2022). Effects of dietary addition of perilla frutescens seeds on the content of polyunsaturated fatty acids in egg yolk of gallus domesticus. Pak. J. Zool. 54, 161–166. 10.17582/journal.pjz/20201203131248

[B20] Feng J. Lu M. Y. Wang J. Zhang H. J. Qiu K. Qi G. H.. (2021). Dietary oregano essential oil supplementation improves intestinal functions and alters gut microbiota in late-phase laying hens. J. Anim. Sci. Biotechnol. 12, 72. 10.1186/s40104-021-00600-3 34225796 PMC8259136

[B21] Griel A. E. Kris-Etherton P. M. (2006). Beyond Saturated Fat: The importance of the dietary fatty Acid profile on cardiovascular disease. Nutr. Rev. 257, 257–262. 10.1111/j.1753-4887.2006.tb00208.x 16770946

[B22] Grigoleit H. G. Grigoleit P. (2005). Pharmacology and preclinical pharmacokinetics of peppermint oil. Phytomedicine. 12, 612–616. 10.1016/j.phymed.2004.10.007 16121523

[B23] Han X. P. Song Y. Huang R. M. Zhu M. Q. Li M. Y. Requena T.. (2023). Anti-inflammatory and gut microbiota modulation potentials of flavonoids extracted from passiflora foetida fruits. Foods. 12, 2889. 10.3390/foods12152889 37569158 PMC10417441

[B24] Huang C. G. Han X. L. Yang Z. P. Chen Y. L. Rengel Z. (2020). Sowing methods influence soil bacterial diversity and community composition in a winter wheat-Summer maize rotation system on the loess plateau. Front. Microbiol. 11, 192. 10.3389/fmicb.2020.00192 32132987 PMC7040079

[B25] Hughes R. L. Marco M. L. Hughes J. P. Keim N. L. Kable M. E. (2019). The role of the gut microbiome in predicting response to diet and the development of precision nutrition models-part I: overview of current methods. Adv. Nutr. 10, 953–978. 10.1093/advances/nmz022 31225589 PMC6855943

[B26] Kang C. H. Molagoda I. M. N. Choi Y. H. Park C. Moon D. O. Kim G. Y. (2018). Apigenin promotes TRAIL-mediated apoptosis regardless of ROS generation. Food. Chem. Toxicol. 111, 623–630. 10.1016/j.fct.2017.12.018 29247770

[B27] Karadaolu Z. Zsoy B. Lmez M. Aydin Z. D. Ahin T. (2018). The effects of drinking water supplemented with essential oils on performance, egg quality and egg yolk fatty acid composition in laying hens. Acta Vet. Eurasia. 44, 85–92. 10.26650/actavet.2018.410397

[B28] Kaya A. Kaya H. Macit M. Celebi S. Esenbuga N. Yoruk M. A.. (2013). Effects of dietary inclusion of plant extract mixture and copper into layer diets on egg yield and quality, yolk cholesterol and fatty acid composition. Kafkas Univ. Vet. Fak. 19, 673–679. 10.9775/kvfd.2013.8644

[B29] Khedr N. F. (2015). Protective effect of mirtazapine and hesperidin on cyclophosphamide-induced oxidative damage and infertility in rat ovaries. Exp. Biol. Med. 240, 1682–1689. 10.1177/1535370215576304 25787947 PMC4935352

[B30] Kim W. H. Lillehoj H. S. (2019). Immunity, immunomodulation, and antibiotic alternatives to maximize the genetic potential of poultry for growth and disease response. Anim. Feed Sci. Techn. 250, 41–50. 10.1016/j.anifeedsci.2018.09.016

[B31] Lee B. K. Kim J. S. Ahn H. J. Hwang J. H. Kim J. M. Lee H. T.. (2010). Changes in hepatic lipid parameters and hepatic messenger ribonucleic acid expression following estradiol administration in laying hens (Gallus domesticus). Poult. Sci. 89, 2660–2667. 10.3382/ps.2010-00686 21076105

[B32] Ley R. E. (2016). Prevotella in the gut: choose carefully. Nat. Rev. Gastroen. Hepatol. 13, 69–70. 10.1038/nrgastro.2016.4 26828918

[B33] Liu X. T. Lin X. Zhang S. Y. Guo C. Q. Li J. Mi Y. L.. (2018). Lycopene ameliorates oxidative stress in the aging chicken ovary via activation of Nrf2/HO-1 pathway. Aging. 10, 2016–2036. 10.18632/aging.101526 30115814 PMC6128425

[B34] Lordelo M. Fernandes E. Bessa R. J. B. Alves S. P. (2017). Quality of eggs from different laying hen production systems, from indigenous breeds and specialty eggs. Poult. Sci. 96, 1485–1491. 10.3382/ps/pew409 27811323

[B35] Masquio D. Piano A. D. Campos R. Sanches P. L. Carnier J. Corgosinho F. C.. (2015). Reduction in saturated fat intake improves cardiovascular risks in obese adolescents during interdisciplinary therapy. Int. J. Clin. Pract. 69, 560–570. 10.1111/ijcp.12573 25296762

[B36] Miranda J. M. Anton X. Redondo-Valbuena C. Roca-Saavedra P. Rodriguez J. A. Lamas A.. (2015). Egg and egg-derived foods: effects on human health and use as functional foods. Nutrients. 7, 706–729. 10.3390/nu7010706 25608941 PMC4303863

[B37] Moyano A. L. Pituch G. Breemen V. Mansson J. E. Givogri M. I. (2013). Levels of plasma sulfatides C18: 0 and C24: 1 correlate with disease status in relapsing-remitting multiple sclerosis. J. Neurochem. 127, 600–604. 10.1111/jnc.12341 23777394

[B38] Mukhopadhya I. Hansen R. El-Omar E. M. Hold G. L. (2012). IBD—what role do Proteobacteria play? Nat. Rev. Gastro. Hepat. 9, 219–230. 10.1038/nrgastro.2012.14 22349170

[B39] Nadia R. Hassan R. A. Qota E. M. Fayek H. M. (2008). Effect of natural antioxidant on oxidative stability of eggs and productive and reproductive performance of laying hens. Int. J. Poult. Sci. 6, 134–150. 10.3923/ijps.2008.134.150

[B40] Nys Y. Gautron J. Garcia-Ruiz J. M. Hincke M. T. (2004). Avian eggshell mineralization: biochemical and functional characterization of matrix proteins. C. R. Palevol. 3, 549–562. 10.1016/j.crpv.2004.08.0029512888

[B41] Peng M. J. Huang T. Yang Q. L. Peng S. Jin Y. X. Wang X. S. (2022). Dietary supplementation Eucommia ulmoides extract at high content served as a feed additive in the hens industry. Poult. Sci. 101, 101650–101650. 10.1016/j.psj.2021.101650 35121531 PMC8814652

[B42] Rahman A. Bayram I. Gultepe E. E. (2021). Effect of mentha on performance, haematological and biochemical parameters in laying hens. S. Afr. J. Anim. Sci. 51, 221–230. 10.4314/sajas.v51i2.10

[B43] Rakonjac S. Bogosavljevic-Boskovic S. Pavlovski Z. Skrbic Z. Doskovic V. Petrovic M. D.. (2014). Laying hen rearing systems: a review of major production results and egg quality traits. Worlds Poult. Sci. J. 70, 93–104. 10.1017/S0043933914000087

[B44] Rattanawut J. Pimpa O. Yamauchi K. E. (2018). Effects of dietary bamboo vinegar supplementation on performance, eggshell quality, ileal microflora composition, and intestinal villus morphology of laying hens in the late phase of production. Anim. Sci. J. 89, 1572–1580. 10.1111/asj.13080 30151990

[B45] Rizzo A. Roscino M. T. Binetti F. Sciorsci R. L. (2012). Roles of reactive oxygen species in female reproduction. Reprod. Domest. Anim. 47, 344–352. 10.1111/j.1439-0531.2011.01891.x 22022825

[B46] Shin N. R. Whon T. W. Bae J. W. (2015). Proteobacteria: microbial signature of dysbiosis in gut microbiota. Trends. Biotechnol. 33, 496–503. 10.1016/j.tibtech.2015.06.011 26210164

[B47] Sun J. Liu J. Ren G. Chen X. T. Cai H. H. Hong J. H.. (2022). Impact of purple sweet potato (*Ipomoea batatas L*.) polysaccharides on the fecal metabolome in a murine colitis model. RSC Adv. 12, 11376–11390. 10.1039/D2RA00310D 35425052 PMC9004255

[B48] van den Brand H. Parmentier H. K. Kemp B. (2004). Effects of housing system (outdoor vs. cages) and age of laying hens on egg characteristics. Br. Poult. Sci. 45, 745–752. 10.1080/00071660400014283 15697013

[B49] Varmuzova K. Matulova M. E. Gerzova L. Cejkova D. Gardan-Salmon D. Panhèleux M.. (2015). Curcuma and Scutellaria plant extracts protect chickens against inflammation and Salmonella Enteritidis infection. Poult Sci. 94, 2049–2058. 10.3382/ps/pev190 26188032

[B50] Wang H. Liang S. S. Li X. Y. Yang X. J. Long F. Y. Yang X. (2019). Effects of encapsulated essential oils and organic acids on laying performance, egg quality, intestinal morphology, barrier function, and microflora count of hens during the early laying period. Poult. Sci. 98, 6751–6760. 10.3382/ps/pez391 31347675 PMC8913957

[B51] Wang W. W. Wang J. Zhang H. J. Wu S. G. Qi G. H. (2019). Transcriptome analysis reveals mechanism underlying the differential intestinal functionality of laying hens in the late phase and peak phase of production. BMC Genom. 20, 970. 10.1186/s12864-019-6320-y 31830910 PMC6907226

[B52] Wang Y. Zhang Y. Hou M. Han W. (2022). Anti-fatigue activity of parsley (Petroselinum crispum) flavonoids via regulation of oxidative stress and gut microbiota in mice. J. Funct. Foods. 89, 104963. 10.1016/j.jff.2022.104963

[B53] Ward T. Larson J. Meulemans J. Hillmann B. Lynch J. Sidiropoulos D.. (2017). BugBase predicts organism-level microbiome phenotypes. BioRxiv. 133462. 10.1101/133462

[B54] Yachida S. Mizutani S. Shiroma H. Shiba S. Nakajima T. Sakamoto T.. (2019). Metagenomic and metabolomic analyses reveal distinct stage-specific phenotypes of the gut microbiota in colorectal cancer. Nat. Med. 25, 968–976. 10.1038/s41591-019-0458-7 31171880

[B55] Zhang C. H. Sheng J. Q. Sarsaiya S. Shu F. X. Liu T. T. Tu X. Y.. (2019). The anti-diabetic activities, gut microbiota composition, the anti-inflammatory effects of Scutellaria-coptis herb couple against insulin resistance-model of diabetes involving the Toll-like receptor 4 signaling pathway. J. Ethnopharmacol. 237, 202–214. 10.1016/j.jep.2019.02.040 30807814

[B56] Zhao J. F. Zhang X. Y. Liu H. B. Brown M. A. Qiao S. Y. (2019). Dietary protein and gut microbiota composition and function. Curr. Protein. Pept. Sci. 20, 145–154. 10.2174/1389203719666180514145437 29756574

[B57] Zhou L. Ding X. M. Wang J. P. Bai S. P. Zeng Q. F. Su Z. W.. (2021). Tea polyphenols increase the antioxidant status of laying hens fed diets with different levels of ageing corn. Anim. Nutr. 7, 650–660. 10.1016/j.aninu.2020.08.013 34401543 PMC8342854

