# Correction: Peppermint extract improves egg production and quality, increases antioxidant capacity, and alters cecal microbiota in late-phase laying hens

**DOI:** 10.3389/fmicb.2025.1746506

**Published:** 2026-01-22

**Authors:** Miaomiao Bai, Hongnan Liu, Yihui Zhang, Shanshan Wang, Yirui Shao, Xia Xiong, Xin Hu, Rongyao Yu, Wei Lan, Yadong Cui, Xiangfeng Kong

**Affiliations:** 1 Hunan Provincial Key Laboratory of Animal Nutritional Physiology and Metabolic Process; National Engineering Laboratory for Pollution Control and Waste Utilization in Livestock and Poultry Production; Key Laboratory of Agro-ecological Processes in Subtropical Region; Hunan Provincial Engineering Research Center for Healthy Livestock and Poultry Production; Scientific Observing and Experimental Station of Animal Nutrition and Feed Science in South-Central, Ministry of Agriculture, Institute of Subtropical Agriculture, Chinese Academy of Sciences, Changsha, Hunan, China; 2 College of Biology and Food Engineering, Fuyang Normal University, Fuyang, China

**Keywords:** laying hens, peppermint extract, egg production and quality, antioxidant capacity, cecal microbiota

There was a mistake in Figure 2 as published. The image of g-unclassified_F082 was erroneously duplicated with the image of g-Megamonas. The corrected Figure 2 appears below.

**Figure 2:**
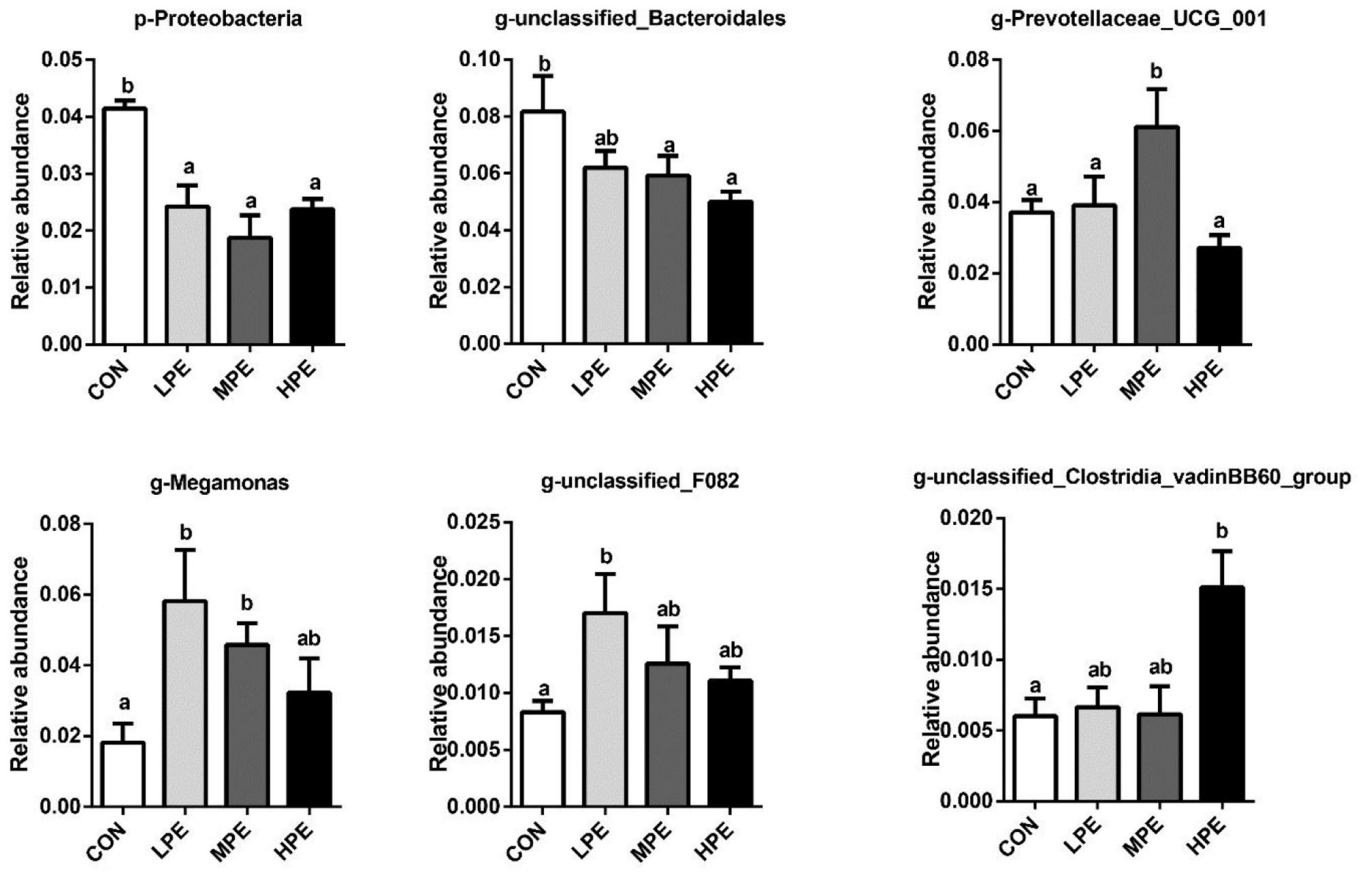
Significantly different cecal microbiota at phylum and genus levels after dietary supplementation with peppermint extract (PE). CON, control group, basal diet; LPE, the control diet + 0.1% PE; MPE, the control diet + 0.2% PE; HPE, the control diet + 0.4% PE. Data are expressed as means ± SEM (*n* = 8). Means within a row with different superscripts are significantly different (*P* < 0.05).

The original version of this article has been updated.

