# Supplementary material for: Peppermint extract improves egg production and quality, increases antioxidant capacity, and alters cecal microbiota in late-phase laying hens
Source: Front Microbiol. 2023 Sep 21;14:1252785. doi: 10.3389/fmicb.2023.1252785 (PMC10552153; doi:10.3389/fmicb.2023.1252785)
Supplement: Supplementary file 1 [file Table_1.docx]

**Supplementary Table S1.** Composition and calculated nutrient levels of the basal diet.

| **Ingredients** | Content, % |
| --- | --- |
| Corn (4.52% crude protein) | 64.55 |
| Soybean meal (43% crude protein) | 22.55 |
| Limestone | 8.89 |
| [Sodium chloride](javascript:;) | 0.32 |
| DL-Methionine | 0.12 |
| L-Lysine | 0.08 |
| Chloride | 0.09 |
| Premix^1^ | 3.40 |
| **Calculated composition** |  |
| Metabolic energy, Mcal/kg | 10.95 |
| Crude protein, % | 14.86 |
| Calcium, % | 3.27 |
| Total phosphorus, % | 0.63 |
| Available phosphorus, % | 0.31 |
| Lysine, % | 0.76 |
| Methionine, % | 0.24 |
| Methionine + cysteine, % | 0.50 |
| **Analyzed composition^3^** |  |
| Gross energy (kcal/kg) | 3722 |
| Dry matter | 91.50 |
| Crude protein | 15.86 |
| Calcium | 3.68 |
| Total phosphorous | 0.46 |
| Neutral detergent fiber | 10.66 |
| Acid detergent fiber | 4.54 |
| Lysine | 0.79 |
| Threonine | 0.70 |
| Methionine | 0.17 |
| Methionine + cysteine | 0.41 |

^1^Provided per kilogram of diet: vitamin A, 50,000 IU; vitamin D3, 3,0000 IU; vitamin E, 400 mg; vitamin K, 40 mg; vitamin B1, 40 mg; vitamin B2, 120 mg; vitamin B6, 70 mg; biotin, 2.5 mg; folic acid, 18 mg; niacin, 550 mg; pantothenic acid, 280 mg; Cu, 12 mg; Fe, 80 mg; Mn, 120 mg; I, 1.0 mg; Se, 0.4 mg; Zn, 85mg.

^2^The nutrient levels were calculated values. Metabolic energy was calculated according to the Table of Feed Composition and Nutritional Value in China (2021).

^3^Dry matter (method GB/T 6435-2014), crude protein (method GB/T 6432-1994), calcium (method GB/T 13885-2017), phosphorus (method GB/T 6437-2002), neutral detergent fiber and acid deter-gent fiber (method NY/T 1459-2022), amino acids (Lysine, Threonine, Methionine and Cysteine, method GB/T 18246-2000) were determined and gross energy was measured by an Isothermal Automatic Heat Meter (5E-AC8018, Kaide Automatic Equipment Changsha Co.,Ltd., Changsha, China).
